# Alpha-Glucosidase and Alpha-Amylase Inhibitory Activities of Novel Abietane Diterpenes from *Salvia africana-lutea*

**DOI:** 10.3390/antiox8100421

**Published:** 2019-09-20

**Authors:** Ninon G.E.R. Etsassala, Jelili A. Badmus, Tesfaye T. Waryo, Jeanine L. Marnewick, Christopher N. Cupido, Ahmed A. Hussein, Emmanuel I. Iwuoha

**Affiliations:** 1 Chemistry Department, University of the Western Cape, Private Bag X17, Bellville 7535, South Africatwaryo@uwc.ac.za (T.T.W.); eiwuoha@uwc.ac.za (E.I.I.); 2 Oxidative Stress Research Unit, Cape Peninsula University of Technology, Symphony Rd. Bellville 7535, South Africa; jabadmus@lautech.edu.ng (J.A.B.); marnewickj@cput.ac.za (J.L.M.); 3 Department of Botany, University of Fort Hare, Private Bag X1314, Alice 5700, South Africa; ccupido@ufh.ac.za; 4 Chemistry Department, Cape Peninsula University of Technology, Symphony Rd. Bellville 7535, South Africa

**Keywords:** diabetes mellitus, Lamiaceae, alpha-glucosidase, alpha-amylase, *Salvia africana-lutea*, terpenoids, cape floristic region

## Abstract

The re-investigation of a methanolic extract of *Salvia africana-lutea* collected from the Cape Floristic Region, South Africa (SA), afforded four new abietane diterpenes, namely 19-acetoxy-12-methoxycarnosic acid (**1**), 3β-acetoxy-7α-methoxyrosmanol (**2**), 19-acetoxy-7α-methoxyrosmanol (**3**), 19-acetoxy-12-methoxy carnosol (**4**), and two known named clinopodiolides A (**5**), and B (**6**), in addition to four known triterpenes, oleanolic, and ursolic acids (**7**, **8**), 11,12-dehydroursolic acid lactone (**9**) and β-amyrin (**10**). The chemical structural elucidation of the isolated compounds was determined on the basis of one and two dimensional nuclear magnetic resonance (1D and 2D NMR), high-resolution mass spectrometry (HRMS), ultra violet (UV), fourier transform infrared (IR), in comparison with literature data. The in vitro bio-evaluation against alpha-glucosidase showed strong inhibitory activities of **8**, **10**, and **7**, with the half inhibitory concentration (IC_50_) values of 11.3 ± 1.0, 17.1 ± 1.0 and 22.9 ± 2.0 µg/mL, respectively, while **7** demonstrated the strongest in vitro alpha-amylase inhibitory activity among the tested compounds with IC_50_ of 12.5 ± 0.7 µg/mL. Additionally, some of the compounds showed significant antioxidant capacities. In conclusion, the methanolic extract of *S. africana-lutea* is a rich source of terpenoids, especially abietane diterpenes, with strong antioxidant and anti-diabetic activities that can be helpful to modulate the redox status of the body and could therefore be an excellent candidate for the prevention of the development of diabetes, a disease where oxidase stress plays an important role.

## 1. Introduction

Diabetes mellitus (DM) is one of the most common metabolic disorders with significant morbidity and mortality rates around the world. It is caused either by deficiency in insulin secretion or degradation of secreted insulin [1], which is the result of cell alterations caused by many internal and external factors, such as obesity, sedentary lifestyle, and oxidative stress [2,3]. However, oxidative stress occurs when the production of free radicals, such as reactive oxygen species (ROS), overwhelms the detoxification capacity of the cellular antioxidant system, resulting in biological damages [4]. It plays a central role in the development of diabetes, such as microvascular and cardiovascular complications [5]. The metabolic abnormalities of diabetes cause mitochondrial superoxide overproduction, which is the main mediator of diabetes tissue damage, insulin resistance, β-cell dysfunction, and impaired glucose tolerance [6].

The prevalence of diabetes reported by the International Diabetes Federation (IFD) indicates that the number of adult diabetic patients globally was 366 million in November 2011, and it is projected to increase to 552 million by the 2030 [7]. In Africa, more than 14 million people have diabetes, accounting for about 4.3% of adults, and it caused about 401,276 deaths in 2012 [8]. Numerous synthetic anti-diabetic agents, such as acarbose, miglitol, sulfonylurea, metformin, and thiozolidinedione, are readily available on the market, but the effectiveness of these products is limited due to non-availability, cost, and high side effects, such as hypoglycaemia, damage to the liver, flatulence, diarrhea, abdominal pain, dropsy, drug-resistance, weight gain, and heart failure [9,10]. Therefore, due to the above-mentioned detrimental effects, there is a great need for developing alternative natural anti-diabetic products with a high safety margin.

Natural plants have been used as a source of medicine since time immemorial for the purpose of curing numerous human afflictions. Plants are known to be the main source of health-promoting substances because they are comprised of secondary metabolites, such as polyphenols, flavonoids, terpenoids, alkaloids, carotenoids, vitamins, and several other constituents, which are responsible for anti-diabetic, antioxidant, anti-hypertensive, and other health promoting effects [11].

*Salvia africana-lutea*, commonly known as *Bruinsalie* or beach sage, is distributed from Namaqualand to the Eastern Cape Province of South Africa (in the South-Western part of South Africa). It is traditionally used for the treatment of different kinds of ailments and/or diseases, such as coughs, sexual debility, mental and nervous conditions, throat inflammation, chronic bronchitis, tuberculosis, influenza, stomach ache, diarrhea, and urticarial [12]. A preliminary phytochemical and biological evaluation of *S. africana-lutea*, collected from Pretoria, South Africa, indicated a great potential of *S. africana-lutea* methanolic extract as a source of abietane diterpenes [13,14]. The ethno-medicinal report of the plant in controlling different symptoms related to ageing diseases justifies the present study to isolate and identify the chemical constituents and explore their in vitro bio-activities. Terpenoids isolated from Lamiaceae, especially abietane diterpenes and some classes of triterpenes, play a crucial role as eco-physiological mediators and are of interest for potential application as therapeutic agents in the treatment of diabetes [15,16]. Recent reports indicate the efficiency of carnosic acid derivatives in controlling the detrimental effects of diabetes by improving glucose and insulin secretion [17], improving glucose homeostasis, or stimulating glucose uptake through intensifying peripheral glucose clearance in tissues, thereby alleviating pathological effects related with the hyperglycaemic state [18]. Carnosol improved diabetes and its complications by modulation of oxidative stress and inflammatory responses [19] and stimulated glucose uptake [20]. Several triterpenes including ursolic acid, oleanolic acid, lupeol, and betulinic acid, have been reported to be potential anti-diabetic candidates with different mechanisms of action [21,22,23].

This work primarily examines the phytochemical isolation of different constituents present in the methanolic extract of *S. africana-lutea* as well as the anti-diabetic and the antioxidant activities of its isolated compounds.

## 2. Experimental Section

### 2.1. General Information

Epigallocatechingallate (EGCG), trolox (6-hydroxyl-2, 5, 7, 8- tetramethylchroman-2-carboxylic acid), and other reagents, including 2,2-azino-bis (3-ethylbenzothiazoline-6-sulfonic acid) (ABTS) diammonium salt, potassium peroxodisulphate, fluorescein sodium salt, 2,2-Azobis (2-methylpropionamidine) dihydrochloride (AAPH), perchloric acid, 2,4,6-tri[2-pyridyl]-s-triazine (TPTZ), iron (III) chloride hexahydrate, copper sulphate, hydrogen peroxide, α-glucosidase (*Saccharomyces cerevisiae*), α-amylase (procaine pancreas), 3,5-dinitro salicylic acid (DNS), p-nitro-phenyl-α-D-glucopyranoside (p-NPG), sodium carbonate (Na_2_CO_3_), sodium dihydrogen phosphate, and di-sodium hydrogen phosphate, secured and purchased from Sigma-Aldrich, Cape town, South Africa.

Organic solvents, methanol (HPLC grade), ethanol, ethyl acetate, and hexane, were supplied by Merck (Cape Town, South Africa). Thin layer chromatography (TLC) as conducted on normal-phase (Merck) Silica gel 60 PF254 pre-coated aluminum plates. Column chromatography was performed using silica gel 60 H (0.040–0.063 mm particle size, Merck, Cape Town, South Africa) and Sephadex LH-20 (Sigma-Aldrich, Cape Town, South Africa).

NMR spectra were recorded on an Avance 400 MHz NMR spectrometer (Bruker, Rheinstetten, Germany) in deuterated chloroform and acetone, using the solvent signals as the internal reference. HRMS analysis was conducted on an Ultimate 3000 LC (Dionex, Sunnyvale, CA, USA) coupled to a Bruker QTOF with an electrospray ionization (ESI) interface working in the positive ion mode. Preparative HPLC was used for further isolation of pure compounds using HPLC methanol and distilled water.

### 2.2. Plant Material

The plant material used in this study was collected from the Cape Flats Nature Reserve, University of the Western Cape, South Africa. A voucher specimen was identified at the Compton Herbarium, Kirstenbosch by Dr. Christopher Cupido (South African National Biodiversity Institute, Kirstenbosch), with herbarium number NBG1465544-0.

### 2.3. Extraction and Purification of Chemical Constituents

The aerial parts of the fresh plant material (2.5 kg) were blended and extracted with methanol (4.5 L) at room temperature (25 °C) for 24 h. The methanol extract was filtered and evaporated to dryness under reduced pressure at 40 °C to yield 97.77 g (3.9%). The total extract (97 g) was applied to a silica gel column (30 × 18 cm) and eluted using a gradient of hexane (Hex) and ethyl acetate (EtOAc) in order of increasing polarity: 94 fractions (500 mL each) were collected and combined according to their TLC profiles to yield 21 fractions, labeled I–XXI.

The main fraction XVII (500 mg) was subjected to a successive silica gel column using a Hex/EtOAc gradient (7:3; 100%) then Sephadex (using 95% aqueous ethanol) to produce 18-acetoxy-12-methoxy carnosic acid (compound **1**, 44.1 mg). The main fraction XIII (1.4 g) was chromatographed on silica gel using a Hex/EtOAc gradient (9:1; 7:3; 100%) then HPLC, using a gradient solvent system of MeOH and de-ionized water (70:30 to 100% MeOH in 45 min), producing compound **3** (R_t_ 23.62 min, 9 mg), compound **4** (R_t_ 25.19 min, 11.17 mg), compound **5** (R_t_ 32.17 min, 46.3 mg), and compound **6** (R_t_ 12.75 min, 40 mg). Main fraction XI was subjected to a successive silica gel column, under the same condition, then HPLC to produce compound 2 (R_t_ 19.67 min, 9.9 mg).

Main fraction X was subjected to a silica gel column under the same condition then chromatographed on a sephadex column (5 % aqueous ethanol) to produce compound **8** (34.6 mg), compound **7** (9.6 mg), compound **9** (12.5 mg), and compound **10** (54 4 mg).

### 2.4. Alpha-Glucosidase Inhibitory Activity

Alpha-glucosidase inhibitory activity of the isolated compounds was carried out according to the standard method, with a slight modification [24]. In a 96-well plate, the reaction mixture containing 50 μL of phosphate buffer (100 mM, pH = 6.8), 10 μL alpha-glucosidase (1 U/mL), and 20 μL of varying concentrations of isolated compounds was pre-incubated at 37 °C for 15 min. Next, 20 μL of p-NPG (5 mM) was added as a substrate and incubated further at 37 °C for 20 min. The reaction was stopped by adding 50 μL of sodium carbonate Na_2_CO_3_ (0.1 M). The absorbance of the released p-nitrophenol was measured at 405 nm using a Multiplate Reader (Multiskan thermo scientific, version 1.00.40, Vantaa, Finland). Acarbose at various concentrations was included as a standard. Each experiment was performed in triplicates. The results were expressed as a percentage inhibition, which was calculated using Formula (1).

Inhibitory activity (%) = (1 − A/B) ×100(1)

where A is the absorbance in the presence of the test substance and B is the absorbance of the control.

### 2.5. Alpha-Amylase Inhibitory Activity

The alpha-amylase inhibitory activity of the extract and fractions was carried out according to the standard method, with a slight modification [24]. In a 96-well plate, the reaction mixture containing 50 μL of phosphate buffer (100 mM, pH = 6.8), 10 μL alpha amylase (2 U/mL), and 20 μL of varying concentrations of isolated compounds was pre-incubated at 37 °C for 20 min. Next, 20 μL of 1% soluble starch (100 mM phosphate buffer pH 6.8) was added as a substrate and incubated further at 37 °C for 30 min. A volume of 100 μL of the color reagent (DNS) was added and then boiled for 10 min. The absorbance of the resulting mixture was measured at 540 nm using a Multiplate Reader (Multiskan thermo scientific, version 1.00.40). Acarbose at various concentrations was used as a standard. Each experiment was performed in triplicates. The results were expressed as the percentage inhibition, which was calculated using Formula (2).

Inhibitory activity (%) = (1 − A/B) ×100(2)

where A is the absorbance in the presence of the test substance and B is the absorbance of the control.

### 2.6. Antioxidant Assays

#### 2.6.1. Ferric-Ion Reducing Antioxidant Power (FRAP) Assay

The FRAP assay was carried out in accordance with the method described previously [25]. Absorbance was measured at 593 nm. l-Ascorbic acid was used as a standard, and the results were expressed as µmole ascorbic acid equivalents per milligram of dry weight (μM AAE/g DW) of the test samples.

#### 2.6.2. Trolox Equivalent Absorbance Capacity (TEAC) Assay

The total anti-oxidant activity of the test sample was measured using previously described methods [26]. Absorbance was read at 734 nm at 25 °C in a plate reader, and the results were expressed as μmole Trolox equivalents per milligram of dry weight (μM TE/g DW) of the test samples.

#### 2.6.3. Oxygen Radical Absorbance Capacity (ORAC) Assay

ORAC assay was done according to the previous method [27]. ORAC values were expressed as micromoles of trolox equivalents (TE) per milligram of the test sample. Samples without a perfect curve were further diluted, and the dilution factors were used in the calculations of such samples.

### 2.7. Statistical Analysis

All the measurements were done in triplicate and IC_50_ values were calculated using GraphPad Prism 5 version 5.01 (Graph pad software, Inc., La Jolla, CA, USA.) statistical software.

## 3. Results and Discussion

### 3.1. Chemical Characterization of the Isolated Compounds

**Figure 1:**
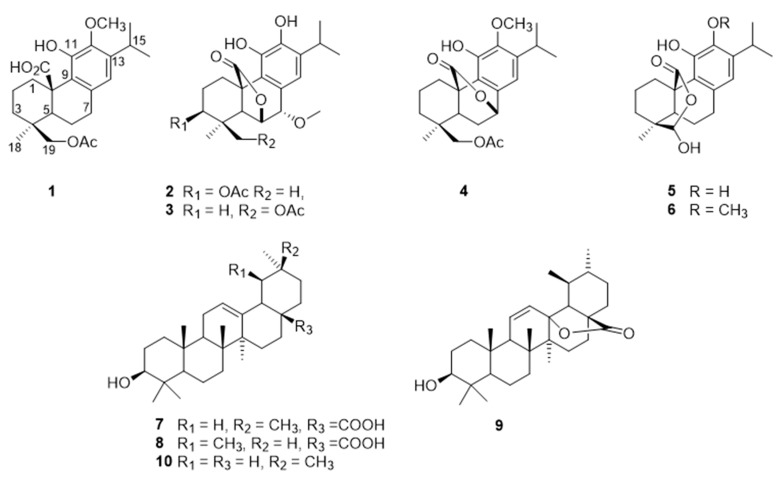
Chemical structures of the isolated compounds (**1**–**10**) from *S. africana-lutea*.

Chromatographic purification of a methanolic extract of *S. africana-lutea* was done using different techniques, including semi prep-HPLC yielded pure terpenoids (Figure 1), four of which were reported for the first time.

**Figure 2:**
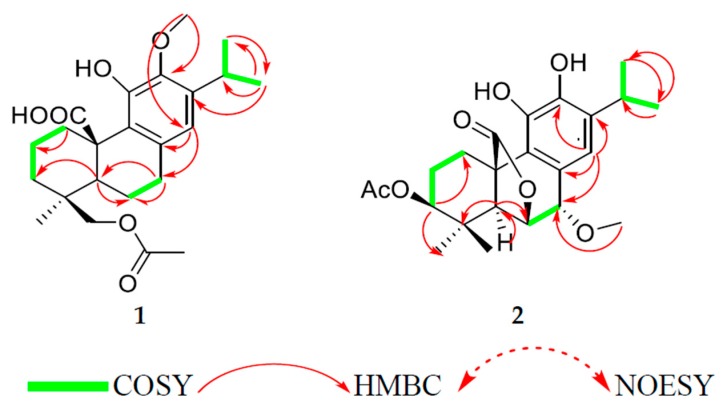
Key ^1^H−^1^H correlation spectroscopy (COSY), heteronuclear multiple bond correlation (HMBC), and nuclear Overhauser effect spectroscopy (NOESY) correlations of **1** and **2**.

Compound **1** was isolated as amorphous yellowish-brown powder. The high-resolution mass spectrometry (HRMS) data indicated a molecular ion peak at [M]^+^ 403.2115 *m*/*z*, suggesting a possible chemical formula of C_23_H_32_O_6_. Its IR spectrum exhibited bands at 1725 and 3447 cm^−1^ forester and hydroxyl groups. The UV spectrum showed two absorptions at 210 and 280 nm. The ^1^H NMR spectra (Table 1, Supplementary Materials) showed signals of three methyls at 1.19 ppm (*d*, *J* = 6.8 Hz), 1.17 (*d*, *J* = 6.8 Hz), and 1.06 (s); an aromatic proton at 6.46 (s); an oxygenated methylene proton at 3.97, 4.24 (*d*/each, *J* = 11.4 Hz); a multiplet signal at 3.17 (*sept*, *J* = 6.8 Hz); in addition to acetoxyl and a methoxyl groups at 1.86, 3.75. The ^13^C NMR, distortionless enhancement by polarization transfer (DEPT-135), and heteronuclear multiple quantum coherence (HSQC) spectra indicated the presence of 23 carbons (Table 2) classified as four methyls, including a methoxy (61.1), two methylene, six methines, one of which was aromatic (117.8), and eight quaternary carbons, including a carbonyl (181.7) and five aromatic carbons (126.8, 133.8, 148.9, 143.7, and 139.6) in addition to the acetoxyl group (172.5, 20.7). The NMR data indicated an abietane diterpene similar to carnosic acid, previously isolated from the same source [14], with the only difference being the presence of extra acetoxyl and methoxyl groups. The methoxyl group was allocated at C-12 from the heteronuclear multiple bond correlation (HMBC) spectra, which showed correlations (among others) between the methoxyl protons and C-12 (143.7). The acetoxyl group was allocated at C-19 due to the presence of a methylene signal at 3.97 and 4.24, and both protons showed HMBC correlations with the acetoxyl’s carbonyl group, C-4/C-5, and C-3. Other 2D data [heteronuclear multiple bond correlation (HMBC) and nuclear overhauser effect spectroscopy (NOESY)] as shown in Figure 2 confirmed the structure of compound **1** as 19-acetoxy-12-methoxycarnosic acid. The absolute configuration of the compound is proposed to belong to the normal abietane skeleton based on a biosynthetic basis, because normal abietane diterpene derivatives were previously isolated from the same source and directly related to the isolated compound [14,28].

The HRMS of compound **2** showed the molecular ion peak at [M]^+^ 417.1907 *m*/*z*, corresponding to the molecular formula of C_23_H_30_O_7_. Its IR spectrum exhibited bands at 1727 and 3447 cm^−1^ for ester and hydroxyl groups. The UV spectrum showed two peaks at 212 and 287 nm. Analysis of the ^1^H NMR data (Table 1) showed the presence of an isopropyl group [a proton at *δ*_H_ = 3.04 (*sept*, *J* = 6.5 Hz); two methyls at 1.24 (d/both, *J* = 6.5 Hz)]; an aromatic proton (*δ*_H_ = 6.79, s); three separated protons attached to three oxygenated carbons at 4.66 (*dd, J* = 3.7, 12.1 Hz), 4.74 (*d*, *J* = 3.2 Hz), and 4.29 (*d*, *J* = 3.2 Hz); in addition to four methyl signals at 1.01 and 0.99 (s each), 2.07 (s, acetoxy), and 3.66 (s, methoxy). The ^13^C NMR, DEPT-135 and HSQC showed 23 carbons classified as four methyls at (16.2, 26.7, 22.3, 22.4), an acetoxy (21.2), a methoxy (58.4), two methylene groups (25.3, 24.3), six methines, (one of which was aromatic), nine quaternary carbons, including two carbonyls (170.7, 178.1), and five aromatics (126.6, 123.4, 141, 141.8, and 134.7). According to HSQC, the proton resonating at *δ*_H_ 4.66 attached to carbon at 74.0 showed HMBC correlations with carbons at 174 (CO acetoxy), 77.4 (C-7), 51.5 (C-5), and 46.5 (C-10), which indicates the presence of a lactone ring at position six. Additionally, the proton at 4.29 (H-7) showed HMBC correlations with carbons at 74.0 (C-6), 126.6 (C-8), 123.4 (C-9), and 120.4 (C-14) as shown in Table 2. On the other hand, the spectroscopic data of compound **2** showed a close similarity with methoxyrosmanol, the only difference being the presence of an extra acetoxyl signal (2.07/170.7). The position of the acetate group was located at C-3 (from HMBC and NOESY correlations). Other 2D spectra confirmed the structure of compound **2** as 3β-acetoxy-7α-methoxyrosmanol [29,30,31].

Compound **3** showed NMR data similar to compound **2**. The ^1^H NMR showed the absence of the C-3 methine signal at 4.7 and the appearance of the signal of methylene protons (4.05 s) attached to an acetoxyl group (from HMBC spectra). The acetoxyl group is attached to C19 because the NOESY and HMBC correlations showed cross-peaks between the acetoxy methyl/C-19, in addition to CH_2_-19/C_3_; C_4_ and C_5_. Other 2D data confirmed the structure of compound **3** as 19-acetoxy-7α-methoxyrosmanol [28].

The NMR of compound **4** (Table 1 and Table 2) showed a close similarity with carnosol [32] and compounds **2** and **3**, except for the absence of the C-6α(OH)/C-7α(OH) system appearing at the C-20/C-7α lactone ring, in addition to a methoxy group, which was located at C-12, as shown in Figure 1. The HMBC spectra showed a correlation between H6/C7; C5; C4; C8 and H7/C6; C8; C13; C9. Other 2D data confirmed the structure of compound **4** as 19-acetoxy-12-methoxy carnosol.

Compound **5** was isolated as a white powder. The HRMS data indicated an ion peak at [M]^+^ 345.1694 *m*/*z*, suggesting a possible chemical formula of C_20_H_28_O_5_. Its IR spectrum exhibited bands at 1675 cm^−1^ for a lactone-carbonyl, as well as at 3500 and 3210 cm^−1^ attributed to hydroxyl groups. The UV spectrum showed two peaks at 208 and 284 nm. The NMR spectra of compound **5** (Table 1 and Table 2) showed similar signals to compound **1**, the difference between them being the absence of the acetoxyl and methoxy1 groups and the appearance of the dioxygenated-carbon signal at 102.8 (C-19), attached to a singlet proton at 5.6, which forms a lactone ring with C-20 (180.1). In particular, the HMBC showed a correlation between a proton at 5.6 (H-19) and the C-20 (180.1)/C-4 (37.0)/C-5 (50.2). Other 2D spectra in comparison with literature data confirmed the structure of compound **5** as clinopodiolide A [33]. On the other hand, compound **6** showed typical NMR signals similar to the ones of compound **5** (Table 1 and Table 2), except for the presence of an extra methoxyl signal, which was placed on C-12 from the HMBC correlations. Other 2D spectra in comparison with literature data confirmed the structure of compound **6** as clinopodiolide B [33]. The occurrence of the lactol moiety at C-19–C-20 is very unusual in nature and it has been recently isolated for the first time from *Salvia clinopodioide*. To the best of our knowledge, these compounds (**5** and **6**) have been isolated for the first time from *S. africana lutea*.

### 3.2. In Vitro Bioactivity

#### 3.2.1. Alpha-Glucosidase and Alpha-Amylase Activities

Alpha-glucosidase is an enzyme located in the brush border of the small intestine epithelium, which catalyzes the breaking down of the reaction of disaccharides and starch to glucose. Glucosidase inhibitors reduce the rate of carbohydrate digestion and delay the carbohydrate absorption from the alimentary tract [29]. Alpha-amylase is one of the main enzymes in humans that is directly involved in the breakdown of starch to simpler sugars [30].

It hydrolyses complex polysaccharides to produce oligosaccharides and disaccharides, which are then hydrolyzed by alpha-glucosidase to monosaccharide, which are absorbed through the small intestines into the hepatic portal vein and increase postprandial glucose levels. The inhibitory mechanisms of these enzymes are characterized by delaying carbohydrate digestion and reducing the rate of glucose absorption [34]. The bio-evaluation of natural resources for the antidiabetic properties has been intensified, and a great deal of research is being carried out to identify plants with potent anti-diabetic activity with emphasis on the inhibition of the two enzymes, alpha-glucosidase and alpha-amylase. In this study, the inhibitory activity of the isolated compounds from *S. africana lutea* was investigated and the results showed that compound **8** demonstrated the highest alpha-glucosidase inhibitory activity with IC_50_ value of 11.3 ± 1.0 µg/mL, followed by compounds **10** and **7** with IC_50_ values of 17.1 ± 1.0 µg/mL and 22.9 ± 2.0 µg/mL, as indicated in Table 3. The IC_50_ value of compound **8** is consistent with the previously reported value of 12.1 ± 1.0 µM [35]. The higher inhibitory activity demonstrated by compound **8** (compared to compound **7**) could be explained by the shift of the C-29 methyl group from C-20 to C-19, which has enhanced the inhibition of the alpha-glucosidase enzyme [35]. In addition, the lowest alpha-glucosidase inhibitory activity demonstrated by compound **10** among the tested triterpenes might be due to the absence of the carboxylic group in its chemical skeleton. Among all the tested abietane diterpenes, only compound **6** demonstrated moderate alpha-glucosidase inhibitory activity, with an IC_50_ value of 81.7 ± 2.1 µg/mL.

Remarkably, compound **7** demonstrated the strongest alpha-amylase inhibitory activity among the tested compounds with an IC_50_ value of 12.5 ± 0.7 µg/mL, followed by compounds **8** and **10** with IC_50_ values of 66.1 ± 2.0 µg/mL and 76.6 ± 2.1 µg/mL, respectively. None of the tested abietane diterpenes showed alpha amylase inhibitory activity, as shown in Table 3.

#### 3.2.2. Antioxidant Activity

The in vitro antioxidant activity of the isolated compounds of the methanolic extract of *S. africana-lutea* were investigated by evaluating their ferric-ion reducing antioxidant power (FRAP), trolox equivalent absorbance capacity (TEAC), and oxygen radical absorbance capacity (ORAC) activities. The TEAC and FRAP are assays based on a single electron transfer (SET) mechanism, in which the antioxidant transfers an electron to the corresponding cationic radical to neutralize it [36], while ORAC is based on a hydrogen atom transfer (HAT) mechanism, in which the antioxidant exhibits the potential health-beneficial roles via transferring hydrogen atom(s) to the reactive species, thereby deactivating them [37]. The results demonstrated that compounds **1** and **5** exhibited strong activity on ORAC (2588.2 ± 10.1; 2357.2 ± 0.1) µM TE/g, respectively. Compounds **5** and **6** showed moderate activities on TEAC (862.2 ± 1.4; 705.5 ± 2.0) µM TE/g, whereas compounds **5** and **2** demonstrated significant inhibitory activity on FRAP (2262.9 ± 11.0; 2200.9 ± 14.2) µM AAE/g when compared to the reference antioxidant epigallocatechingallate (EGCG), as shown in Table 4. Phenolic compounds have been reported to be responsible for the antioxidant activity of numerous plant species by stabilization of radicals by donating electrons or by metal ion complexation, among other mechanisms. Nevertheless, other aspects can be considered, for example, the presence of vicinal hydroxyl groups is essential in a pronounced antioxidant activity [38]. Abietane diterpenes are known for having strong antioxidant activity due to the presence of ortho-dihydroxyl groups/vicinal hydroxyls in the aromatic ring that serve as hydrogen and/or electron donating agents to the corresponding reactive species leading to the formation of the stable quinone derivatives [39]. It has been shown that the phenolic group at the 11 position would be more implicated in the antioxidant activity [40]. In general, phenolic compounds are expected to transfer electrons or donate protons to the reactive radicals because of the resonance stability of the phenoxy radical [41].

The structure-activity relationship (SAR) of compound **5** could be related to the presence of ortho-dihydroxyl groups/vicinal hydroxyls in the aromatic ring as well as the presence of the free hydroxyl groups at the 19 position in its chemical structure, which are responsible for its high activity, demonstrated when compared to compound **6**. However, the substitution of the free OH at the 12 position by a methoxyl group is directly related to the decrease of the activity observed. The presence of an acetoxy group in compound **1** could be responsible for its high capacity of hydrogen atom transfer, demonstrated in the ORAC assay. However, the antioxidant activity of compound **2** demonstrated in FRAP is high because of the high-stress lactone ring, which may open during the course of the chemical reaction leading to an extension of the conjugation and formation of the p-quininoidal structure.

Compound **1**: Yellow, amorphous solid; [α]^28^_D_ +70.43 (0.1, MeOH); UV (MeOH) λ_max_ (log ε) 210 (4.31), 280 (3.79); nm; IR (KBr) ν_max_ 3447, 3320, 2954, 1725, 1570, 1381, 1250, 1039cm^–1^; ^1^H; and ^13^C NMR data, see Table 1 and Table 2; positive-ion high-resolution electrospray ionisation mass spectrometry (HRESIMS) [M−H]^+^ *403.2115* (calcd for C_23_H_32_O_6_, 404.2199).

Compound **2**: Red brownish powder; [α]^28^_D_ +22.92 (0.2, MeOH); UV (MeOH) λ_max_ (log ε) 212 (4.32), 287 (4.09); nm; IR (KBr) ν_max_ 3447, 3300, 2958, 1727, 1575, 1246, 1034 cm^–1^; ^1^H; and ^13^C NMR data, see Table 1 and Table 2; positive-ion HRESIMS [M−H]^+^ *417.1890* (calcd for C_23_H_32_O_6_, 418.1932).

Compound **3**: Brown amorphous powder; [α]^28^_D_ +49.04 (0.07, MeOH); UV (MeOH) λ_max_ (log ε) 210 (4.31), 295 (3.92); nm; IR (KBr) ν_max_ 3450, 2962, 1769, 1634, 1435, 1239, 1034 cm^–1^; ^1^H; and ^13^C NMR data, see Table 1 and Table 2; positive-ion HRESIMS [M−H]^+^ *417.1907* (calcd for C_23_H_32_O_6_, 418.20).

Compound **4**: Yellow amorphous powder; [α]^28^_D_ -53.27 (0.03, MeOH); UV (MeOH) λ_max_ (log ε) 210 (4.31), 281 (4.13); nm; IR (KBr) ν_max_ 3330, 2950, 1750, 1617, 1446, 1243, 1050 cm^–1^; ^1^H; and ^13^C NMR data, see Table 1 and Table 2; positive-ion HRESIMS [M−H]^+^ 401.1609 (calcd for C_23_H_32_O_6_, 402.2042).

## 4. Conclusions

The phytochemical and in vitro bio-activity investigation of the *S. africana-lutea* methanolic extract revealed that this plant is a rich source of abietane diterpenes and triterpenes with significant alpha glucosidase and alpha amylase inhibitory activities, as well as significant antioxidant activity when considering the FRAP, TEAC, ORAC assays. The present work is the first scientific report on *S. africana-lutea*, and the results suggest that the methanolic extract of this plant and/or its individual constituents might become prominent natural therapeutic agents for the inhibition of alpha glucosidase and alpha amylase enzymes and oxidative stress, which both play an important role in the development of diabetic related diseases. Therefore, compounds with high antioxidant and anti-diabetic activities are the most logical choice for reducing diabetes-induced ROS.

## Figures and Tables

**Table 1:** NMR spectroscopic data assignments (400 MHz) for compounds **1**–**6** (δ in ppm, m, J in Hz) in CDCl_3_.

N°	1	2	3	4	5	6
	δ_H_ (*J* in Hz)	δ_H_ (*J* in Hz)	δ_H_ (*J* in Hz)	δ_H_ (*J* in Hz)	δ_H_ (*J* in Hz)	δ_H_ (*J* in Hz)
1	3.47 br d (10.7)	3.28 br d (15.5)	3.22 br d (14.3)	3.22 br d (13.4)	3.34 br d (13.4)	3.42 br d (13.0)
	1.16 *	2.28 ddd (4.2, 14.3, 14.3)	2.06 dd (5.2, 14.1)	1.64 ddd (5.8, 11.0, 13.4)	1.47 ddd (5.4, 13.4, 13.4)	1.46 ddd (5.4, 13.4, 13.4)
2	2.42 m	1.81 m	1.53 ddddd (3.7, 14.0, 14.0, 14.0, 14.0)	1.40 ddd (4.7, 13.3, 13.3)	1.74 *	1.73 *
	1.48 br d (15.2)	1.62 m	1.69 *			
3	1.66 br d (13.7)	4.66 dd (3.7, 12.1)	1.83 br d (14.0)	3.28 d (4.6)	2.3 br d (13.5)	2.3 br d (12.5)
	1.19 *		1.23 d (6.8)	1.64 br d (5.5)	1.23 *	1.21 *
5	1.58 d (12.0)	2.42 s	2.38 s	1.91 *	1.61 br d (12.4)	1.57 br d (13.4)
6	1.47 br d (14.3)	4.74 d (3.2)	4.26 d (3.2)	1.37 d (4.7)	2.11 br d (13.5)	2.11 br d (12.5)
	1.93 br d (16.7)			1.92 d (2.2)	1.31 ddd (5.4, 12.4, 12.4)	1.43 ddd (5.4, 12.4, 12.4)
7	2.75 br d (5.3)	4.29 d (3.2)	4.9 d (3.2)	4.6 d (3.3)	2.77 m	2.8 m
14	6.46 s	6.79 s	6.82 s	6.54 s	6.55 s	6.56 s
15	3.17 sept (6.8)	3.04 sept (6.5)	3.06 sept (7.0)	3.23 sept (7.0)	3.24 sept (7.0)	3.28 sept (7.0)
16	1.19 d (6.8)	1.24 d (6.5)	1.23 d (7.0)	1.22 (7.0)	1.23 d (7.0)	1.2 d (7.0)
17	1.17 d (6.8)	1.24 d (6.5)	1.23 d (7.0)	1.22 (7.0)	1.23 d (7.0)	1.19 d (7.0)
18	1.06 s	1.01 s	1.05 s	1.19 s	1.15 s	1.13 s
19	3.97 d (11.4)	0.99 s	4.05 s	4.16 d (11.8)	5.61 s	5.61 s
	4.24 d (11.4)			4.24 d (11.8)		
OCOCH_3_	1.86 s	2.07 s	2.09 s	2.08 s		
OCH_3_	3.75 s	3.66 s	3.66s	3.76 s		3.86 s

Singlet (s); doublet (d); septuplet (sept); multiplet (m); broad doublet (br d); doublet doublet (dd); * not well defined; doublet doublet doublet (ddd).

**Table 2:** NMR spectroscopic data assignments (100 MHz) for compounds **1**–**6** (δ in ppm) in CDCl_3_.

N°	1	2	3	4	5	6
	^13^C	^13^C	^13^C	^13^C	^13^C	^13^C
1	34.6	25.3	27.1	28.3	35.4	35.0
2	19.7	24.3	18.4	20.9	21.2	21.0
3	37.1	77.5	33.2	28.3	32.5	32.7
4	36.9	36.0	51.0	45.0	37.0	36.9
5	44.3	51.5	50.9	51.8	50.3	50.2
6	20.0	74.0	77.4	21.0	21.6	21.4
7	32.9	77.4	73.9	78.7	30.7	30.9
8	126.8	126.6	126.1	133.7	122.3	123.8
9	133.8	123.4	123.8	119.1	128.0	133.3
10	49.0	46.5	47.0	44.0	49.8	49.4
11	148.9	141.0	142.4	147.9	143.4	149.7
12	143.7	141.8	141.8	143.0	142.1	146.3
13	139.6	134.7	134.8	140.2	133.9	141.8
14	117.8	120.4	119.1	117.8	118.9	118.4
15	26.5	27.4	27.3	26.5	27.2	26.7
16	23.5	22.4	22.2	22.7	22.3	23.5
17	23.5	22.3	22.4	23.5	24.1	23.3
18	27.6	16.2	26.0	25.5	24.1	24.1
19	68.7	26.7	66.2	65.7	102.8	103.3
20	181.7	178.1	178.5	174.3	180.0	180.0
OCOCH_3_	172.5	170.7	170.8	170.9		
OCOCH_3_	20.7	21.2	20.9	20.9		
OCH_3_	61.1	58.4	58.4	61.9		58.6

**Table 3:** Inhibitory activities of the isolated compounds on alpha-glucosidase and alpha-amylase.

Items	Alpha-GlucosidaseIC_50_ (µg/mL)	Alpha-AmylaseIC_50_ (µg/mL)
**1**	NA	NA
**2**	NA	NA
**3**	NA	NA
**4**	NA	NA
**5**	NA	NA
**6**	81.7 ± 2.1	NA
**7**	22.9 ± 2.0	12.5 ± 0.7
**8**	11.3 ± 1.0	66.1 ± 2.0
**9**	85.8 ± 2.3	NA
**10**	17.1 ± 1.0	76.6 ± 2.1
**Acarbose**	610.4 ± 1.0	10.2 ± 0.6

Not active (NA) at the test concentrations. The results are expressed as mean ± SEM for *n* = 3.

**Table 4:** Antioxidant activities of the isolated compounds.

Items	ORAC (µmole TE/g)	TEAC (µmole TE/g)	FRAP (µM AAE/g)
**1**	2588.2 ± 10.1	694.0 ±1.6	1217.4 ± 2.9
**2**	2233.9 ± 8.0	635.7 ± 0.8	2200.9 ± 14.2
**3**	735.4 ± 2.0	124.4 ± 0.6	1440.4 ± 9.1
**4**	559.7 ± 15.2	440.1 ± 1.5	1257.0 ± 6.7
**5**	2357.2 ± 0.1	862.2 ± 1.4	2262.9 ± 11.0
**6**	1502.5 ± 21.2	724.9 ± 1.3	1480.0 ± 3.9
**7**	NA	NA	347.8 ± 3.7
**9**	NA	NA	283.4 ± 3.9
**8**	NA	NA	778.9 ± 6.8
**10**	NA	NA	412.2 ± 13.0
**EGCG**	3976.8 ± 3.8	4146.4 ± 19.8	7525.0 ± 4.9

Not active (NA) at the test concentrations; epigallocatechingallate (EGCG). Trolox equivalent absorbance capacity (TEAC); oxygen radical absorbance capacity (ORAC); ferric-ion reducing antioxidant power (FRAP). The results are expressed as mean ± SEM for *n* = 3.

## Supplementary Materials

The following are available online at https://www.mdpi.com/2076-3921/8/10/421/s1, Supplementary Figures 1–10: ^1^H-NMR (400 MHz, CDCl3) Spectrum, ^13^C-NMR (400 MHz, CDCl3) Spectrum, DEPT-NMR (400 MHz, CDCl3) Spectrum, COSY (400 MHz, CDCl3) Spectrum, HSQC (400 MHz, CDCl3) Spectrum, HMBC (400 MHz, CDCl3) Spectrum, NOESY (400 MHz, CDCl3) Spectrum, HR-MS Spectrum, UV Spectrum and FTIR Spectrum of Compound **1**; Supplementary Figures 11–20: ^1^H-NMR (400 MHz, CDCl3) Spectrum, ^13^C-NMR (400 MHz, CDCl3) Spectrum, DEPT-NMR (400 MHz, CDCl3) Spectrum, COSY (400 MHz, CDCl3) Spectrum, HSQC (400 MHz, CDCl3) Spectrum, HMBC (400 MHz, CDCl3) Spectrum, NOESY (400 MHz, CDCl3) Spectrum, HR-MS Spectrum, UV Spectrum and FTIR Spectrum of Compound **2**; Supplementary Figures 21–28: ^1^H-NMR (400 MHz, CDCl3) Spectrum, ^13^C-NMR (400 MHz, CDCl3) Spectrum, DEPT-NMR (400 MHz, CDCl3) Spectrum, HSQC (400 MHz, CDCl3) Spectrum, HMBC (400 MHz, CDCl3) Spectrum, HR-MS Spectrum, UV Spectrum, FTIR Spectrum of Compound **3**; Supplementary Figures 29–37: ^1^H-NMR (400 MHz, CDCl3) Spectrum, ^13^C-NMR (400 MHz, CDCl3) Spectrum, DEPT-NMR (400 MHz, CDCl3) Spectrum, COSY (400 MHz, CDCl3) Spectrum, HSQC (400 MHz, CDCl3) Spectrum, HMBC (400 MHz, CDCl3) Spectrum, HR-MS Spectrum, UV Spectrum, FTIR Spectrum of Compound **4**.
