# Supplementary material for: Alpha-Glucosidase and Alpha-Amylase Inhibitory Activities of Novel Abietane Diterpenes from Salvia africana-lutea
Source: Antioxidants (Basel). 2019 Sep 20;8(10):421. doi: 10.3390/antiox8100421 (PMC6827013; doi:10.3390/antiox8100421)
Supplement: Supplementary file 1 [file antioxidants-08-00421-s001.pdf]

SUPPLEMENTARY MATERIALS

Alpha-Glucosidase and Alpha-Amylase Inhibitory Activities of Novel Abietane Diterpenes from *Salvia africana-lutea*

Ninon G.E.R. Etsassala [1], Jelili A. Badmus [2], Tesfaye T. Waryo [1], Jeanine L. Marnewick [2], Christopher N Cupido [3], Ahmed A. Hussein [4,*] and Emmanuel I. Iwuoha [1]

Author to whom correspondence should be addressed;

E-mail: mohammedam@cput.ac.za (A.H.); Tel.: +27-21-959-6193; Fax: +27-21-959-3055.

Table of contents

Figure 1: ^{1}H-NMR (400 MHz, CDCl3) Spectrum of Compound **1**
Figure 2: ^{13}C-NMR (400 MHz, CDCl3) Spectrum of Compound **1**
Figure 3: DEPT-NMR (400 MHz, CDCl3) Spectrum of Compound **1**
Figure 4: COSY (400 MHz, CDCl3) Spectrum of Compound **1**
Figure 5: HSQC (400 MHz, CDCl3) Spectrum of Compound **1**
Figure 6: HMBC (400 MHz, CDCl3) Spectrum of Compound **1**
Figure 7: NOESY (400 MHz, CDCl3) Spectrum of Compound **1**
Figure 8: HR-MS Spectrum of Compound **1**
Figure 9: UV Spectrum of Compound **1**
Figure 10: FTIR Spectrum of Compound **1**

Figure 11: ^{1}H-NMR (400 MHz, CDCl3) Spectrum of Compound **2**
Figure 12: ^{13}C-NMR (400 MHz, CDCl3) Spectrum of Compound **2**
Figure 13: DEPT-NMR (400 MHz, CDCl3) Spectrum of Compound **2**
Figure 14: COSY (400 MHz, CDCl3) Spectrum of Compound **2**
Figure 15: HSQC (400 MHz, CDCl3) Spectrum of Compound **2**
Figure 16: HMBC (400 MHz, CDCl3) Spectrum of Compound **2**
Figure 17: NOESY (400 MHz, CDCl3) Spectrum of Compound **2**
Figure 18: HR-MS Spectrum of Compound **2**
Figure 19: UV Spectrum of Compound **2**
Figure 20: FTIR Spectrum of Compound **2**

Figure 21: ^{1}H-NMR (400 MHz, CDCl3) Spectrum of Compound **3**
Figure 22: ^{13}C-NMR (400 MHz, CDCl3) Spectrum of Compound **3**
Figure 23: DEPT-NMR (400 MHz, CDCl3) Spectrum of Compound **3**
Figure 24: HSQC (400 MHz, CDCl3) Spectrum of Compound **3**
Figure 25: HMBC (400 MHz, CDCl3) Spectrum of Compound **3**
Figure 26: HR-MS Spectrum of Compound **3**
Figure 27: UV Spectrum of Compound **3**
Figure 28: FTIR Spectrum of Compound **3**

Figure 29: ^{1}H-NMR (400 MHz, CDCl3) Spectrum of Compound **4**
Figure 30: ^{13}C-NMR (400 MHz, CDCl3) Spectrum of Compound **4**
Figure 31: DEPT-NMR (400 MHz, CDCl3) Spectrum of Compound **4**
Figure 32: COSY (400 MHz, CDCl3) Spectrum of Compound **4**
Figure 33: HSQC (400 MHz, CDCl3) Spectrum of Compound **4**
Figure 34: HMBC (400 MHz, CDCl3) Spectrum of Compound **4**
Figure 35: HR-MS Spectrum of Compound **4**
Figure 36: UV Spectrum of Compound **4**
Figure 37: FTIR Spectrum of Compound **4**

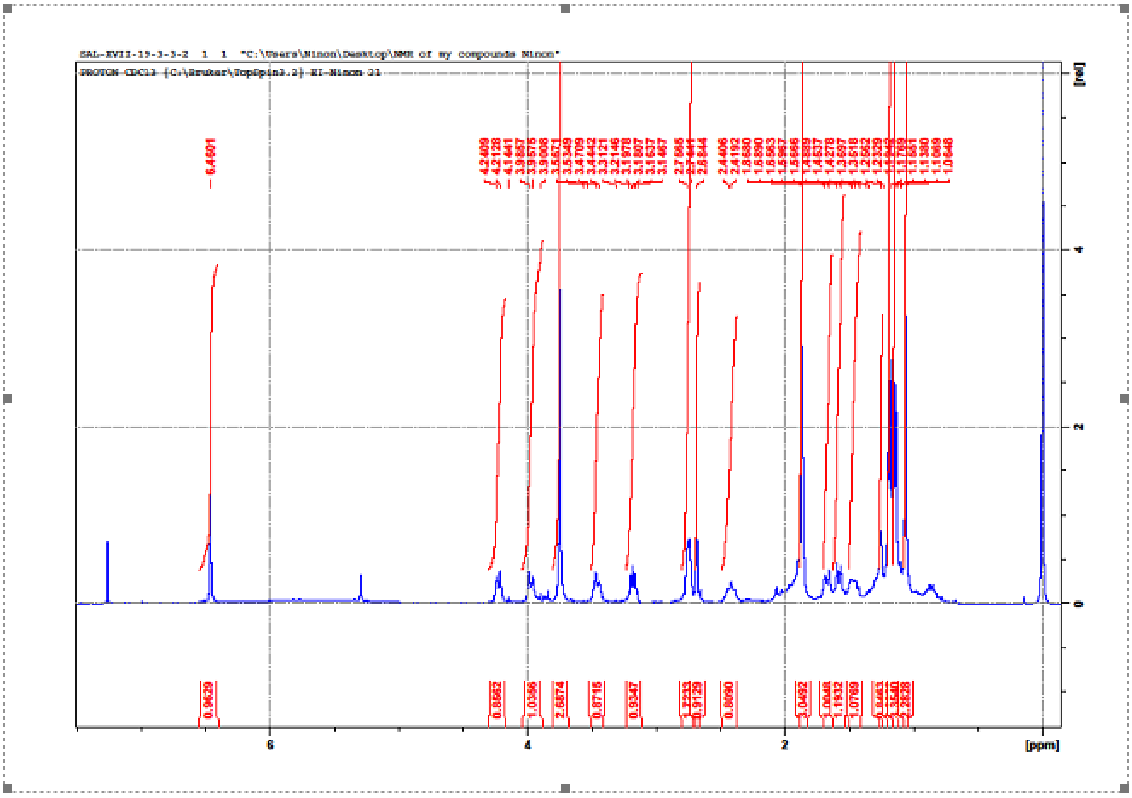

Figure 1: ^{1}H-NMR (400 MHz, CDCl3) Spectrum of Compound **1**

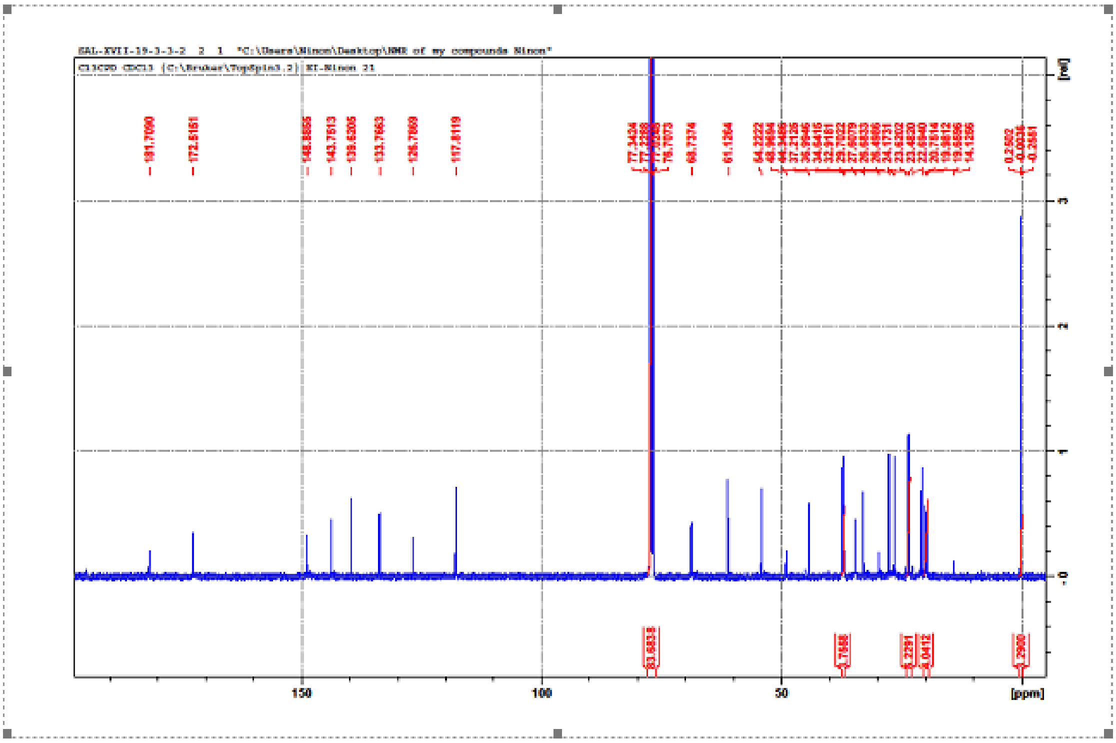

Figure 2: ^{13}C-NMR (400 MHz, CDCl3) Spectrum of Compound **1**

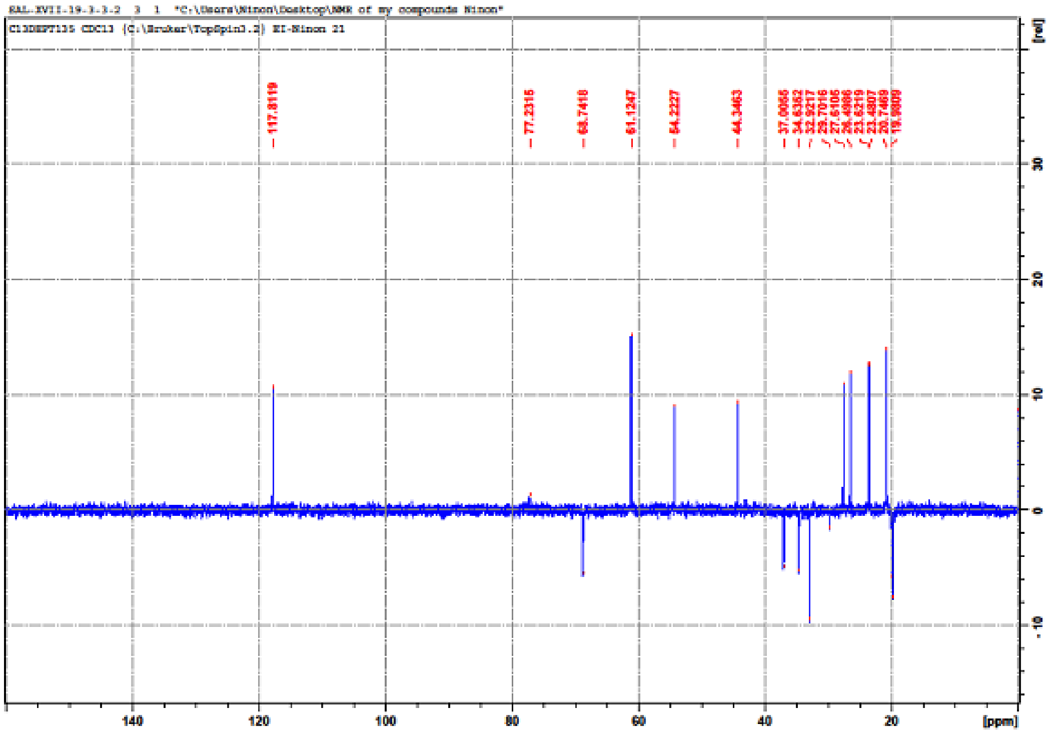

Figure 3: DEPT-NMR (400 MHz, CDCl3) Spectrum of Compound **1**

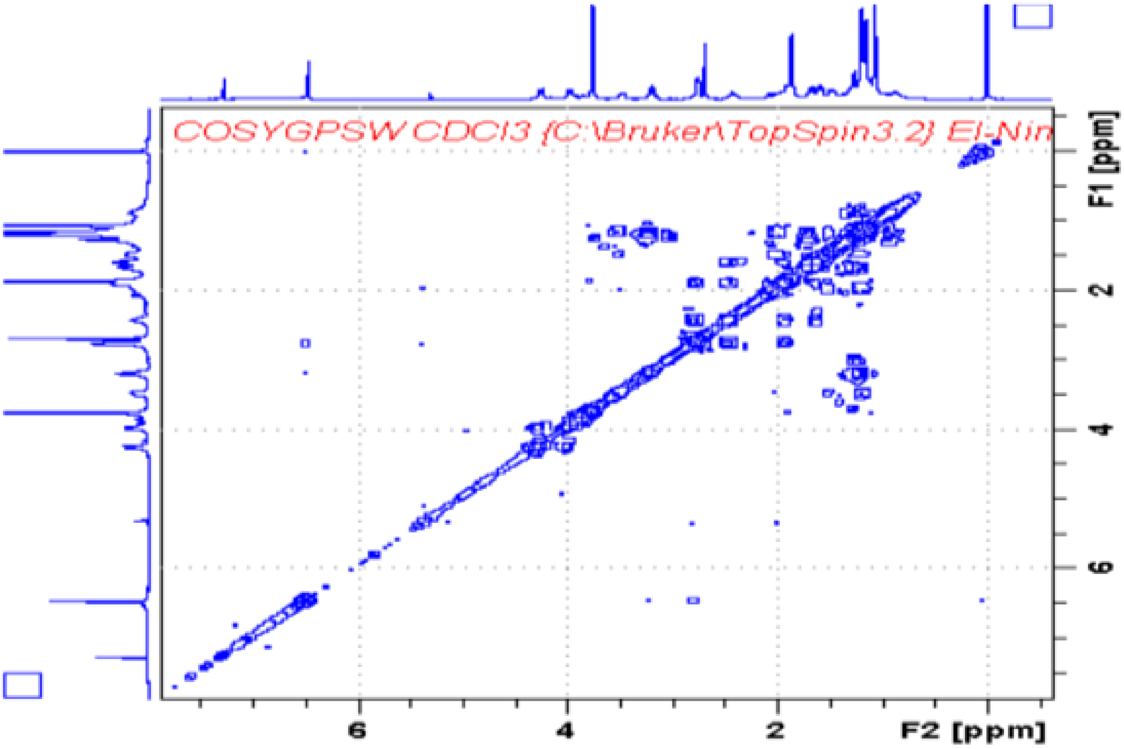

Figure 4: COSY (400 MHz, CDCl3) Spectrum of Compound **1**

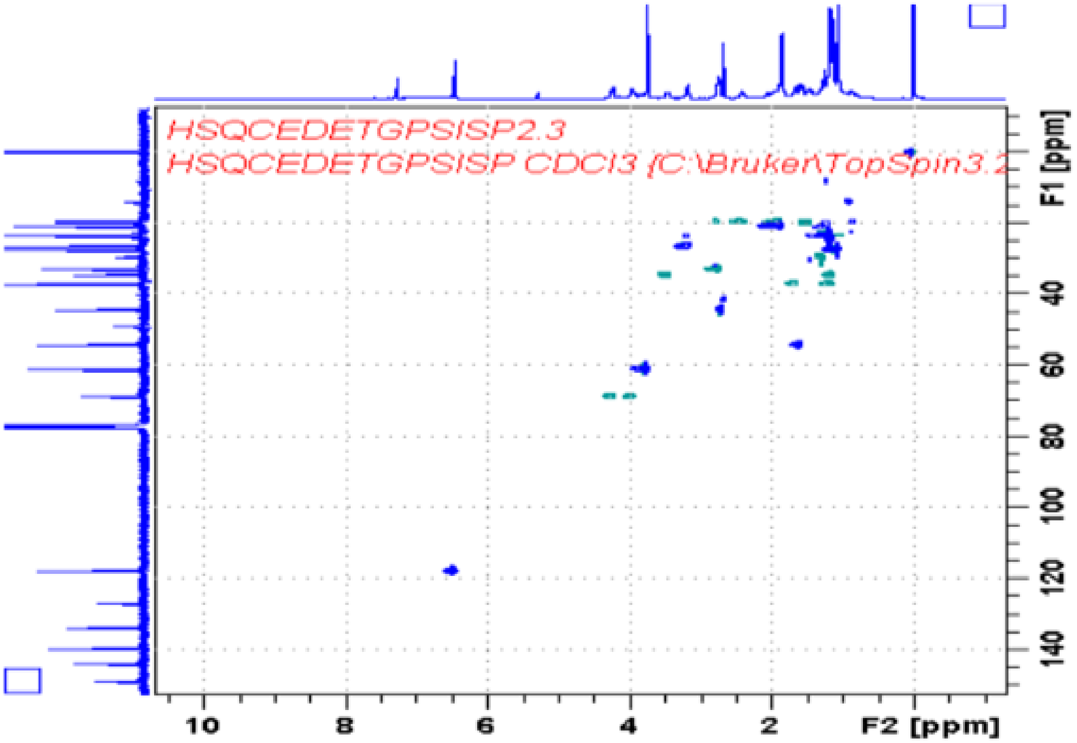

Figure 5: HSQC (400 MHz, CDCl3) Spectrum of Compound **1**

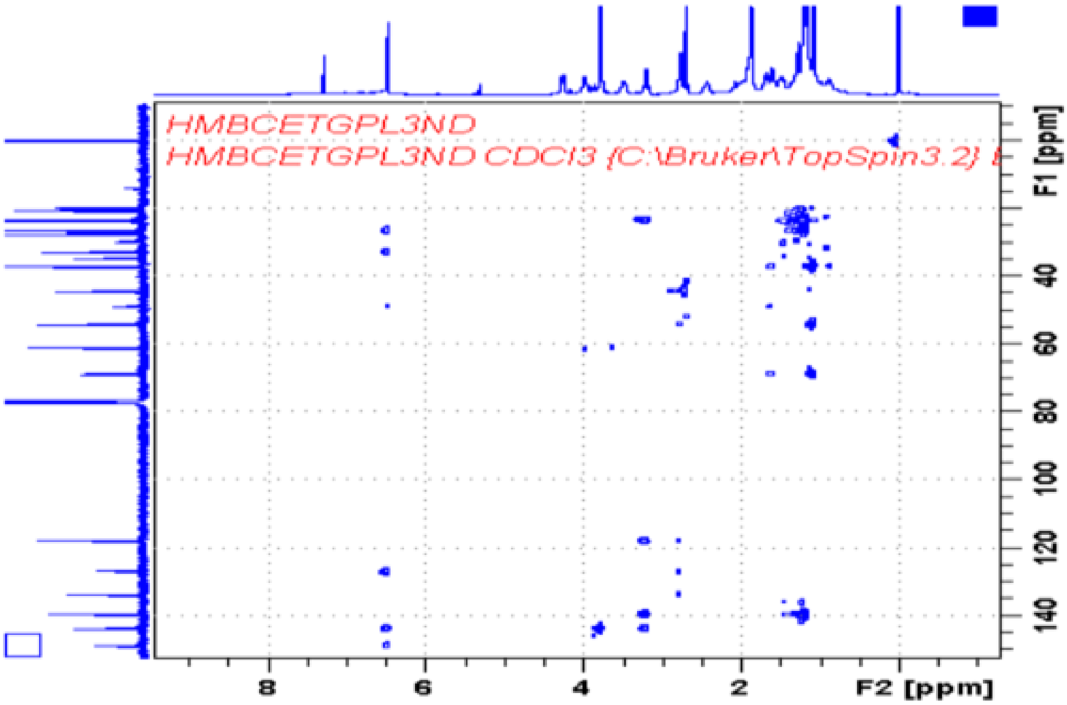

Figure 6: HMBC (400 MHz, CDCl3) Spectrum of Compound **1**

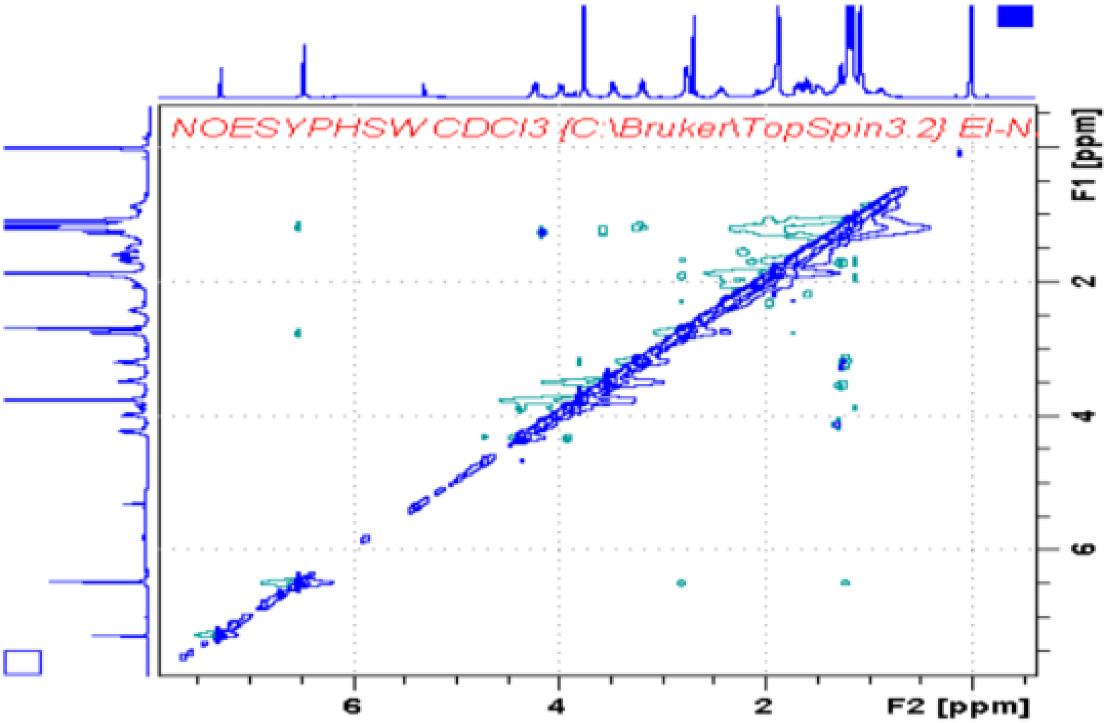

Figure 7: NOESY(400 MHz, CDCl3) Spectrum of Compound **1**

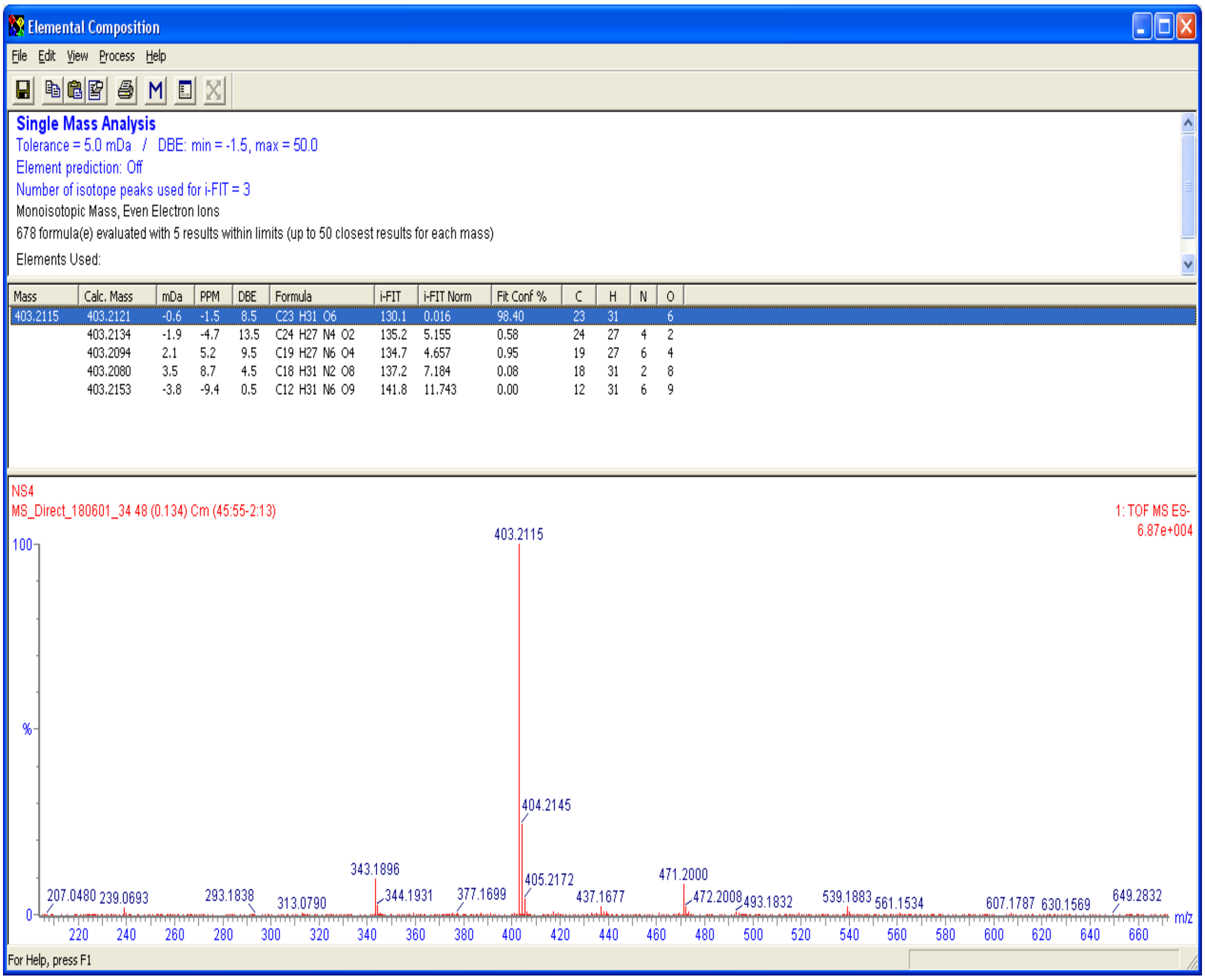

Figure 8: HR-MS spectrum of Compound **1**

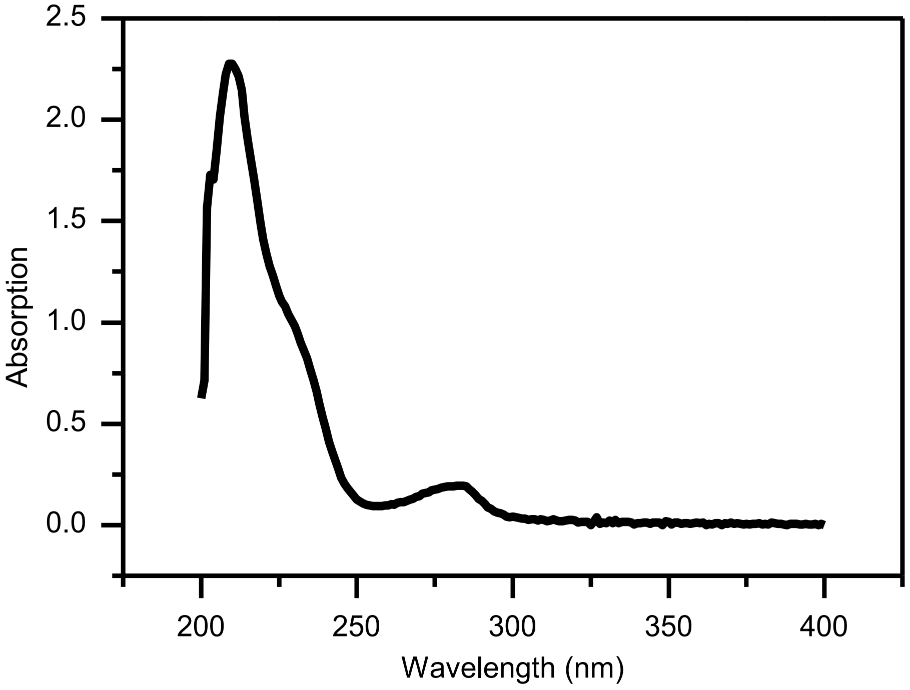

Figure 9: UV spectrum of Compound **1**

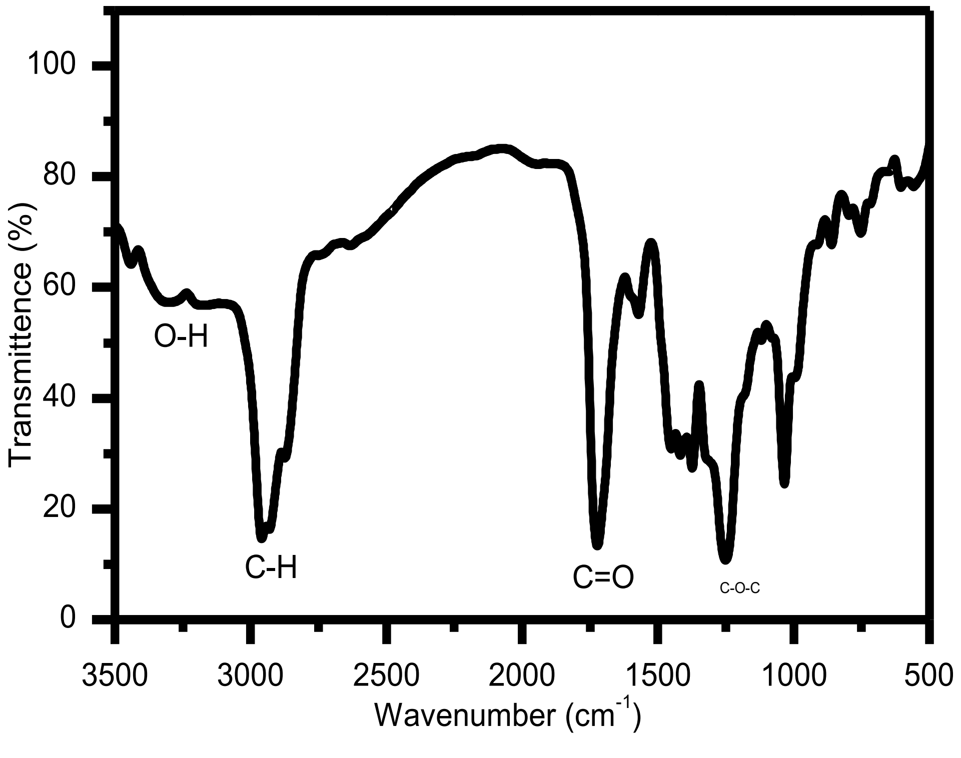

Figure 10: FTIR spectrum of Compound **1**

Compound 2

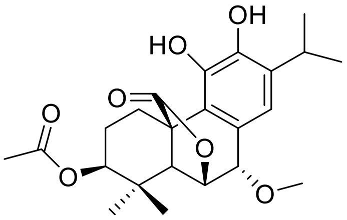

3β-acetoxy-7-methoxy rosmanol

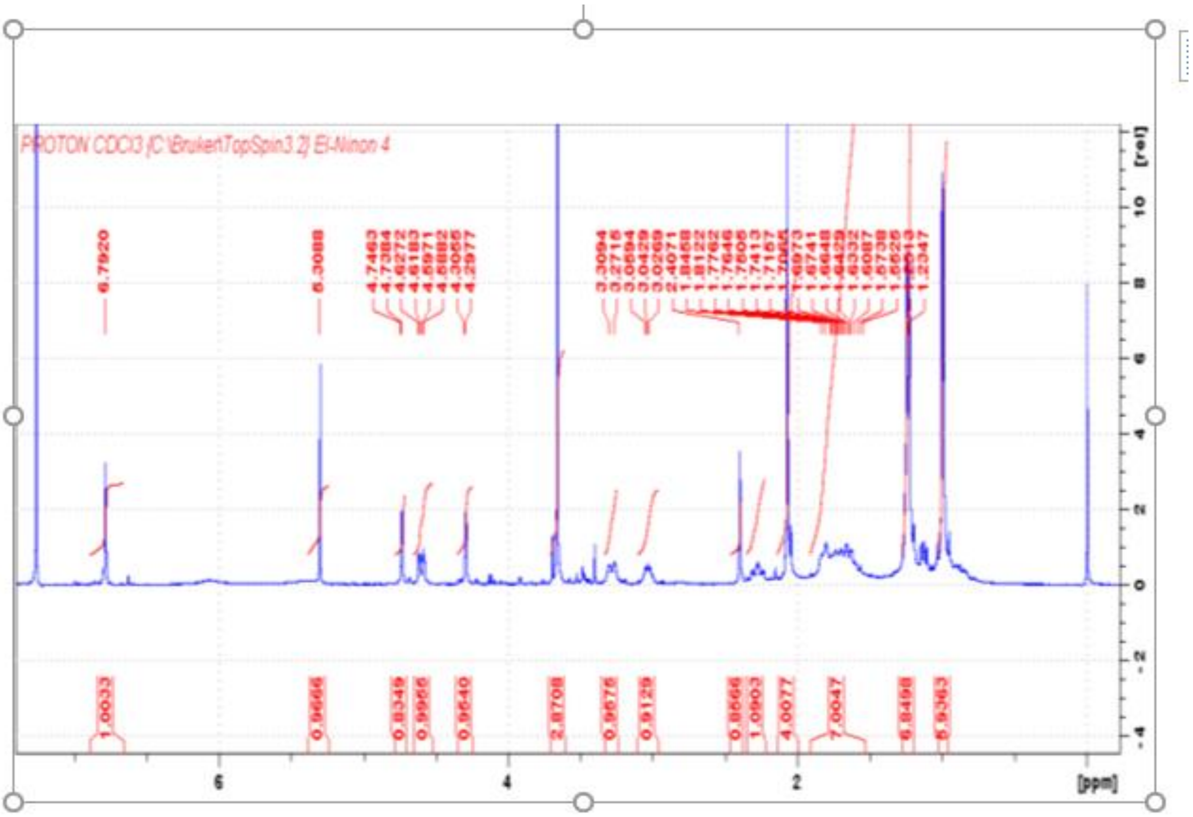

Figure 11: ^{1}H-NMR (400 MHz, CDC13) Spectrum of Compound **2**

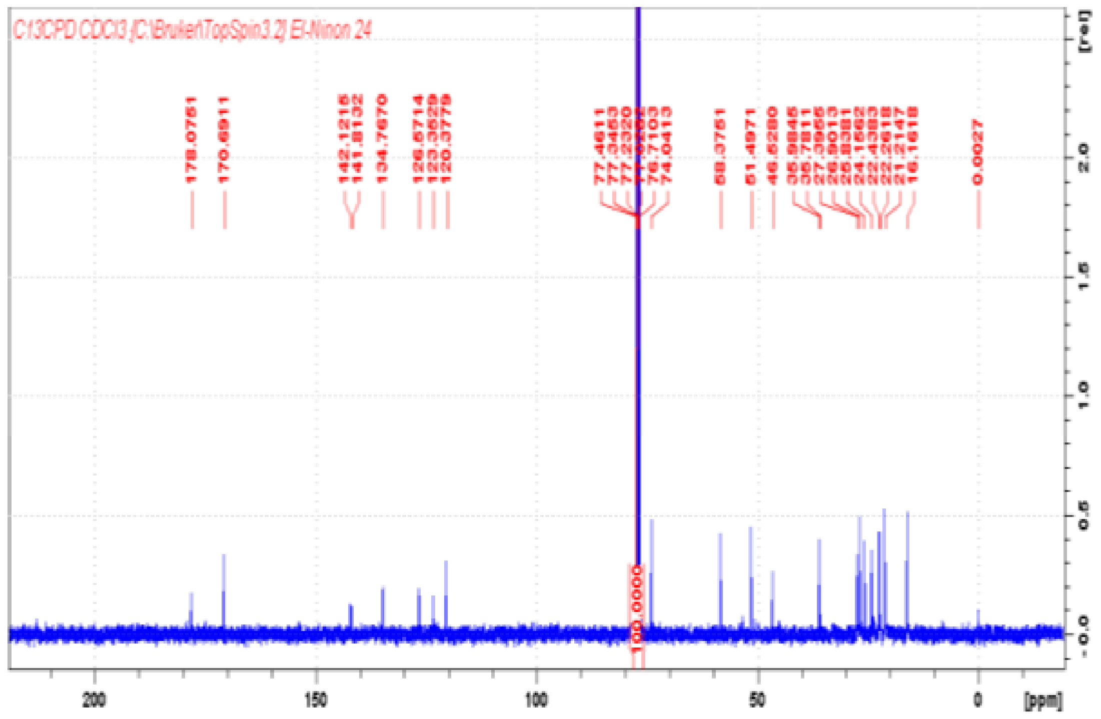

Figure 12: ^{13}C-NMR (400 MHz, CDCl3) Spectrum of Compound **2**

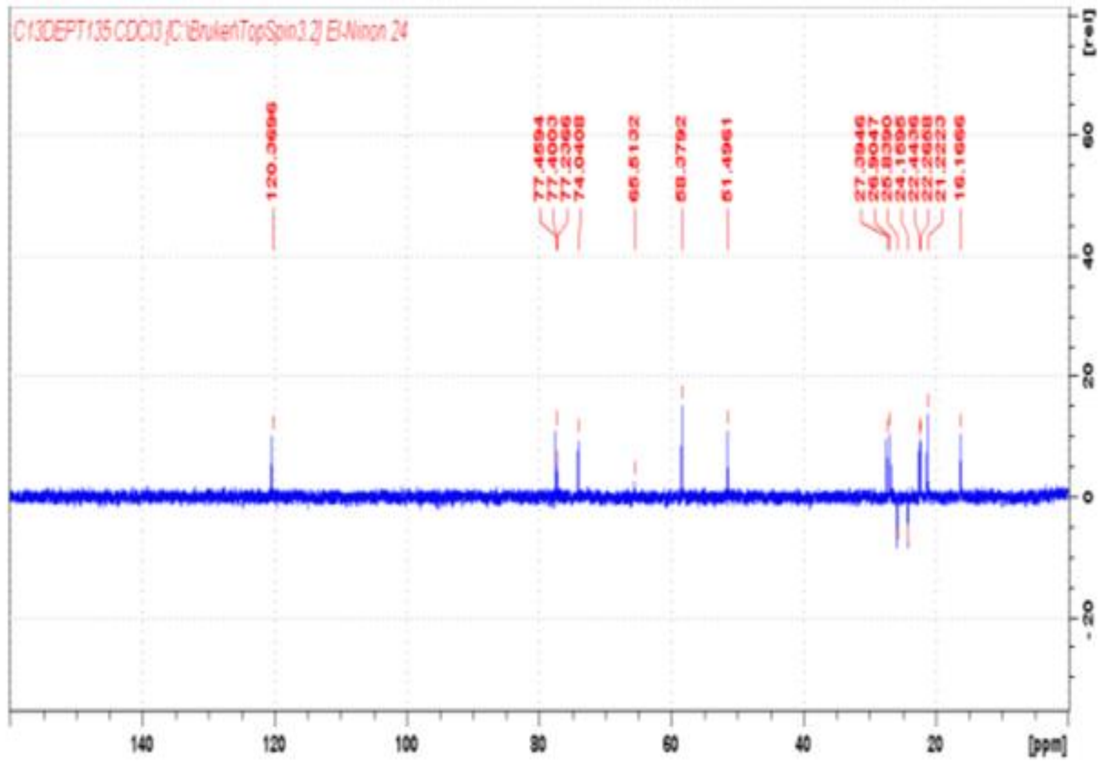

Figure 13: DEPT-NMR (400 MHz, CDCl3) Spectrum of Compound **2**

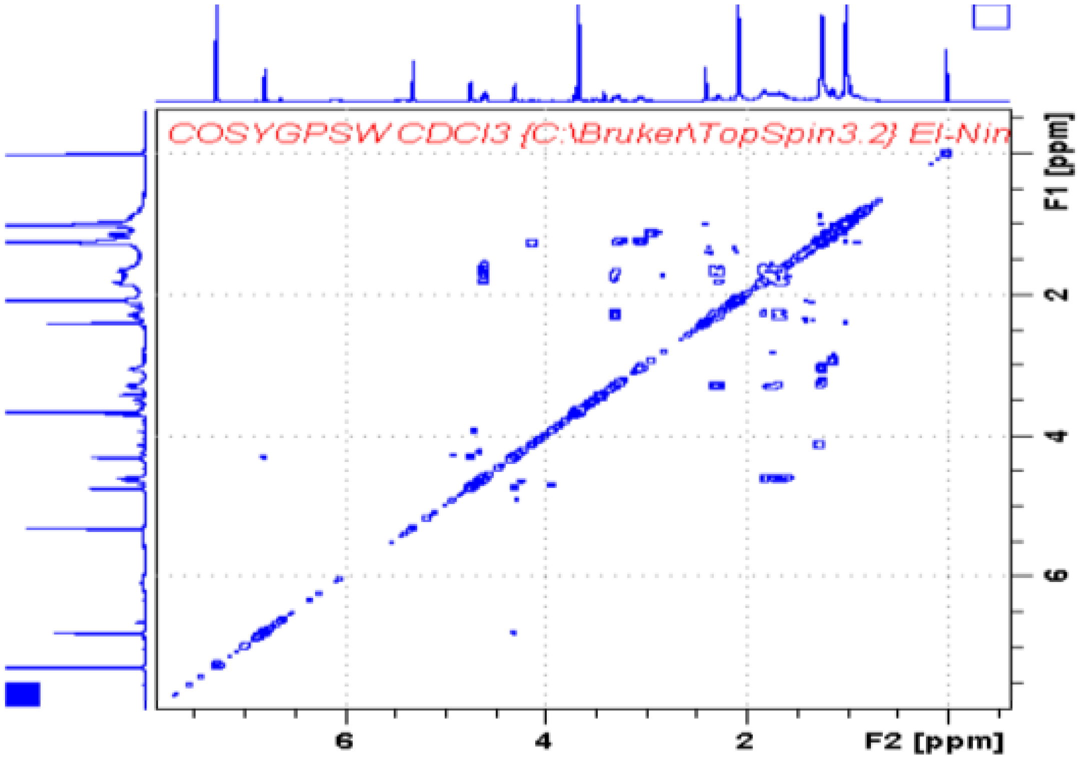

Figure 14: COSY (400 MHz, CDCl3) Spectrum of Compound **2**

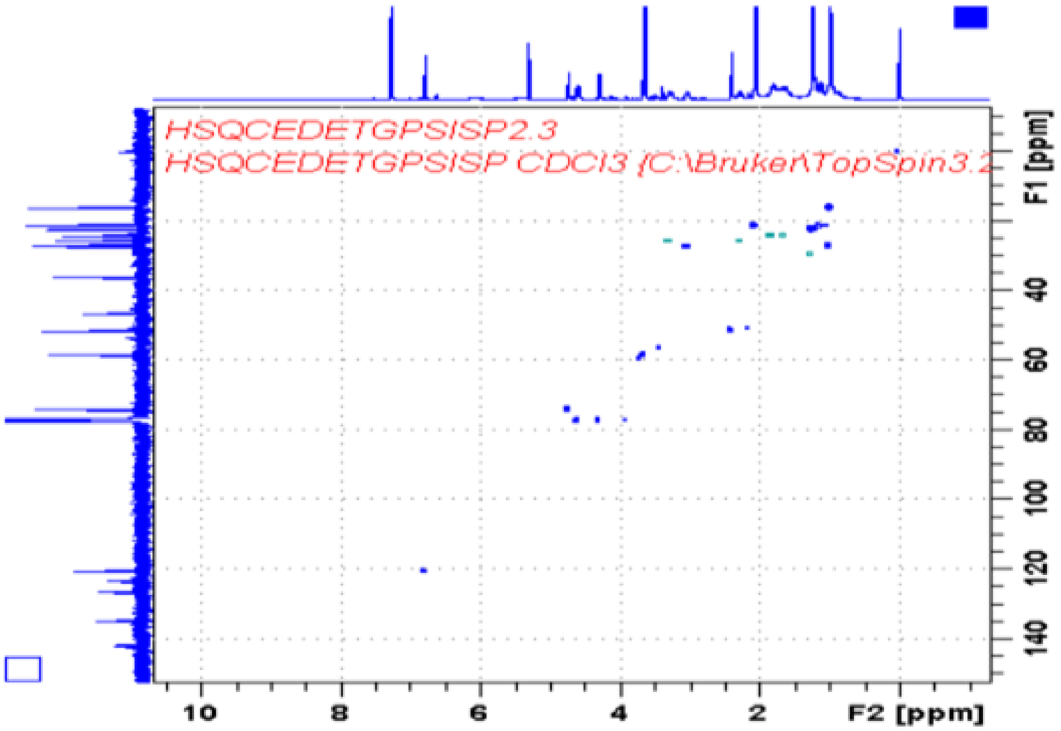

Figure 15: HSQC (400 MHz, CDCl3) Spectrum of Compound **2**

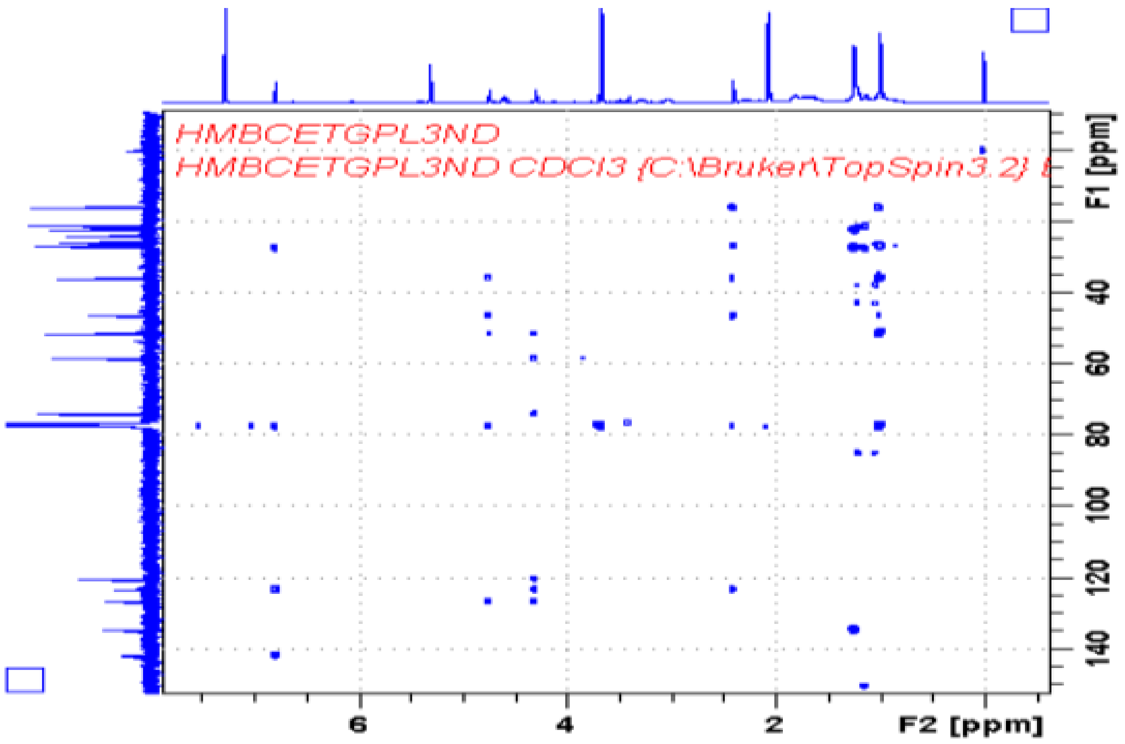

Figure 16: HMBC (400 MHz, CDCl3) Spectrum of Compound **2**

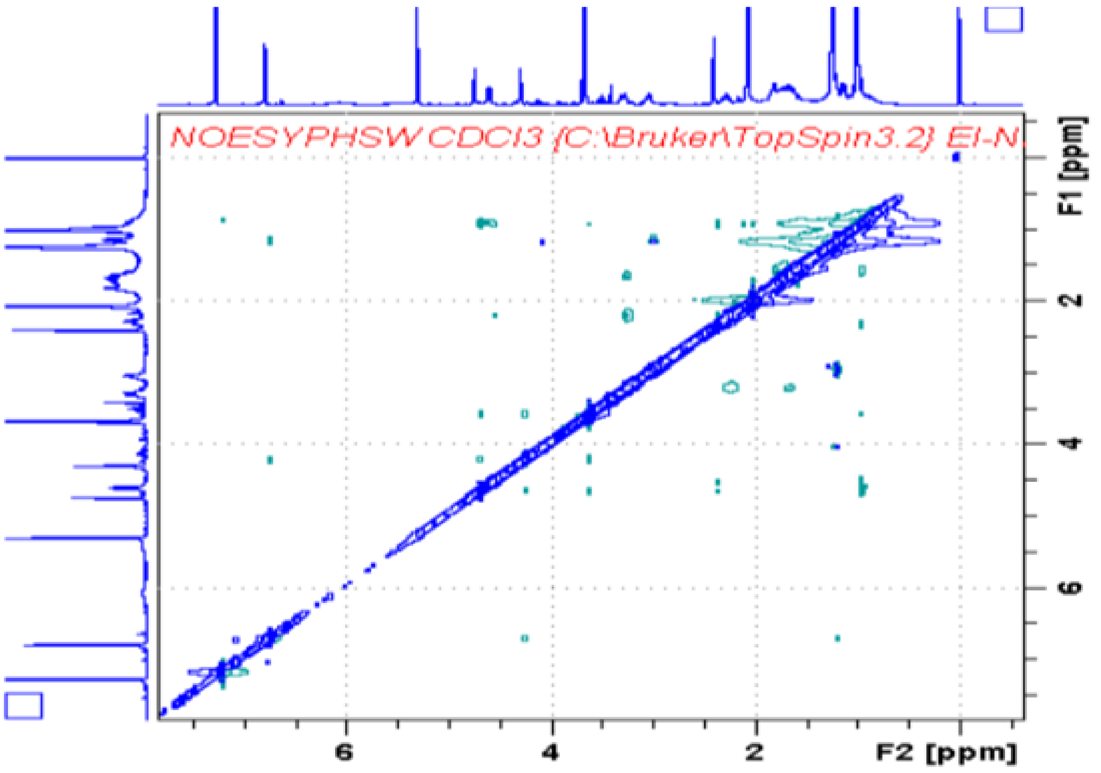

Figure 17: NOESY (400 MHz, CDCl3) Spectrum of Compound **2**

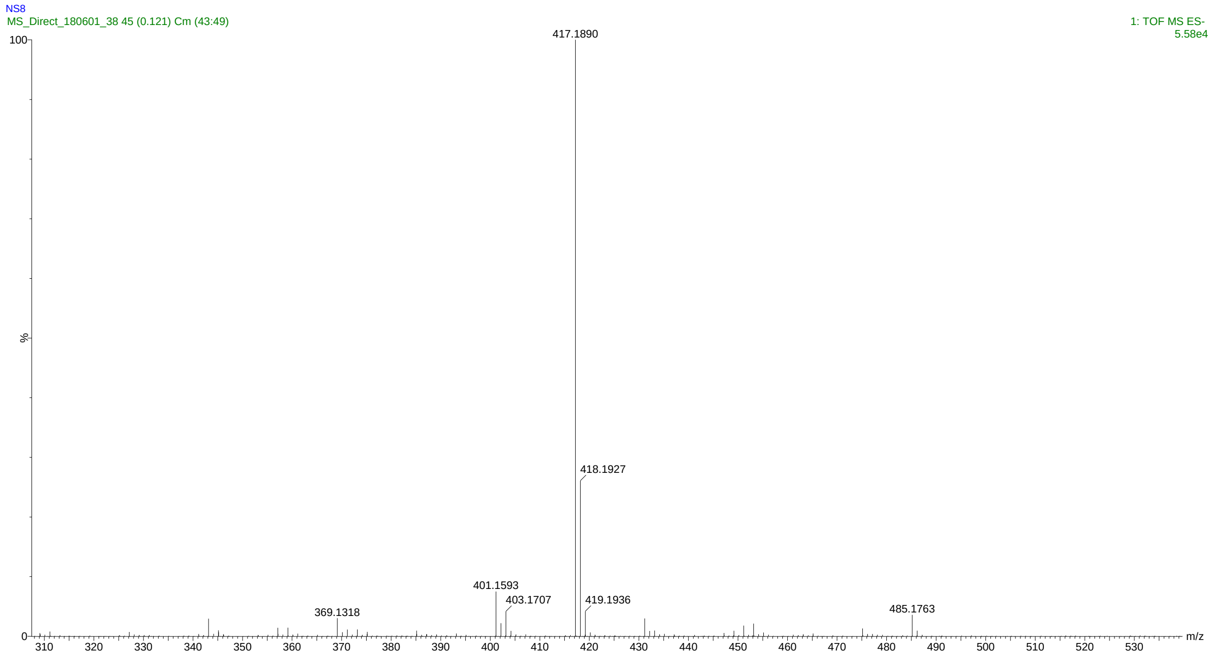

Figure 18: HR-MS spectrum of Compound **2**

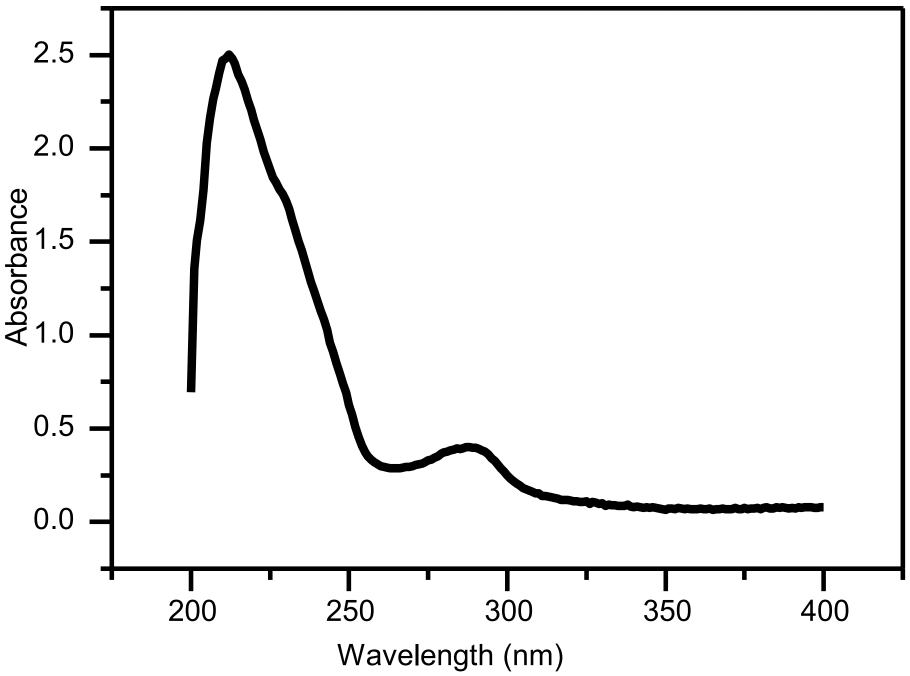

Figure 19: UV spectrum of Compound **2**

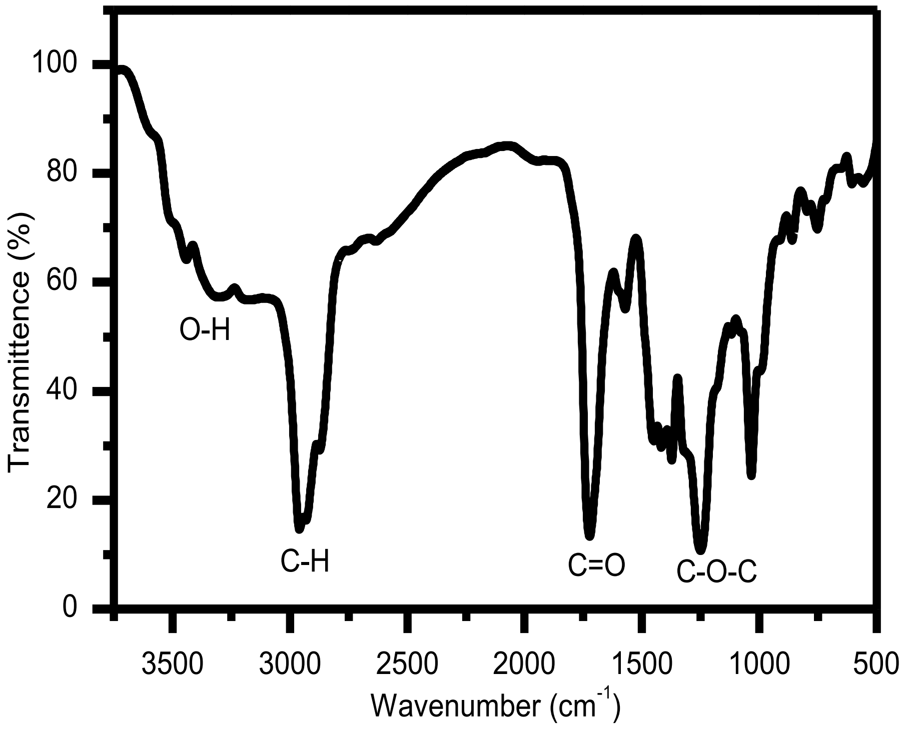

Figure 20: FTIR spectrum of Compound **2**

Compound 3

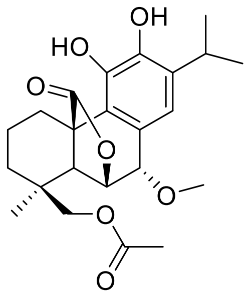

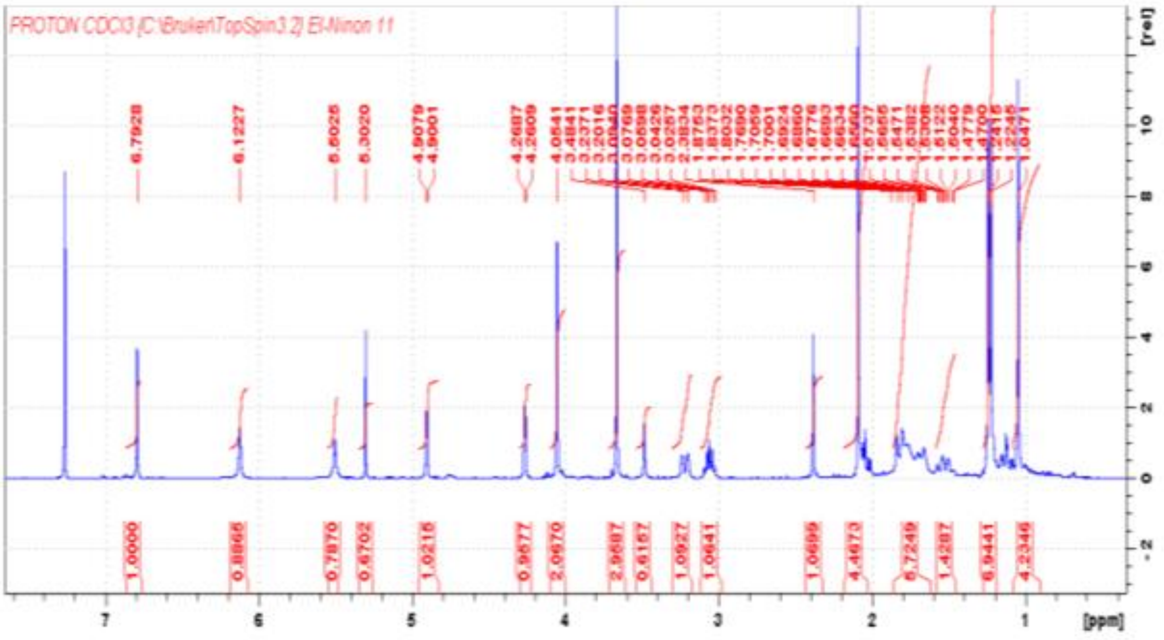

Figure 21: ^{1}H-NMR (400 MHz, CDCl3) Spectrum of Compound **3**

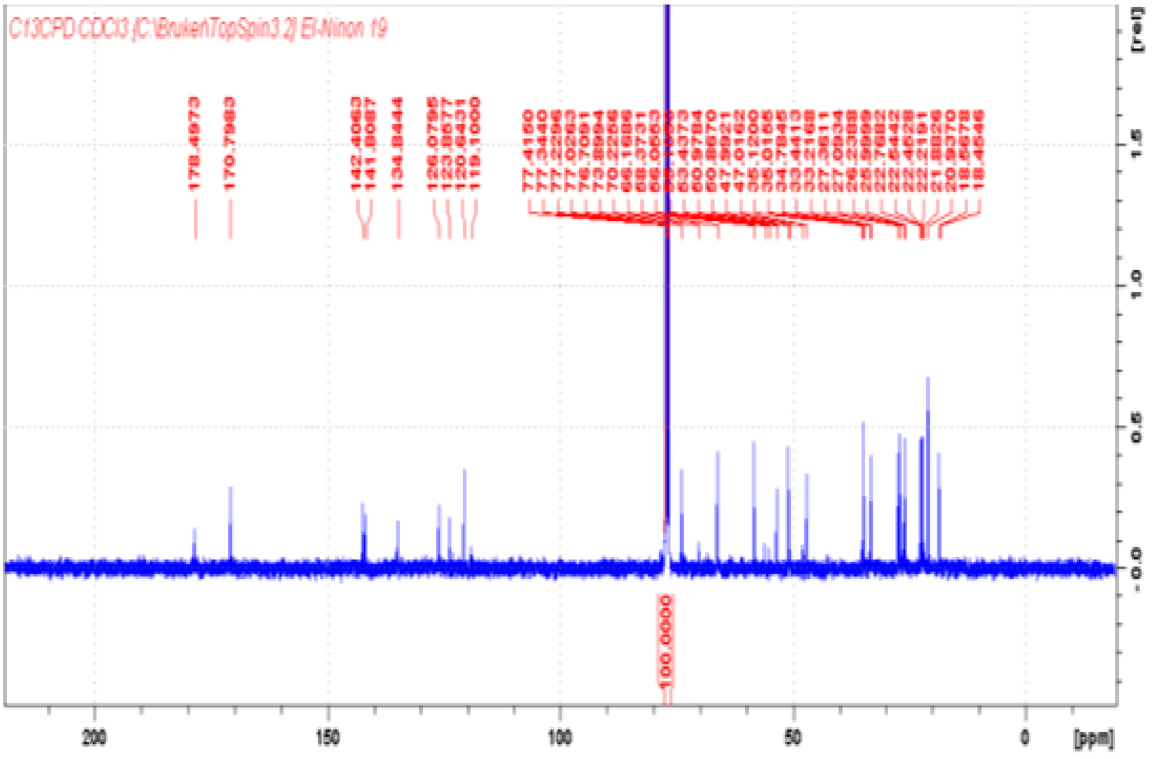

Figure 22: ^{13}C-NMR (400 MHz, CDCl3) Spectrum of Compound **3**

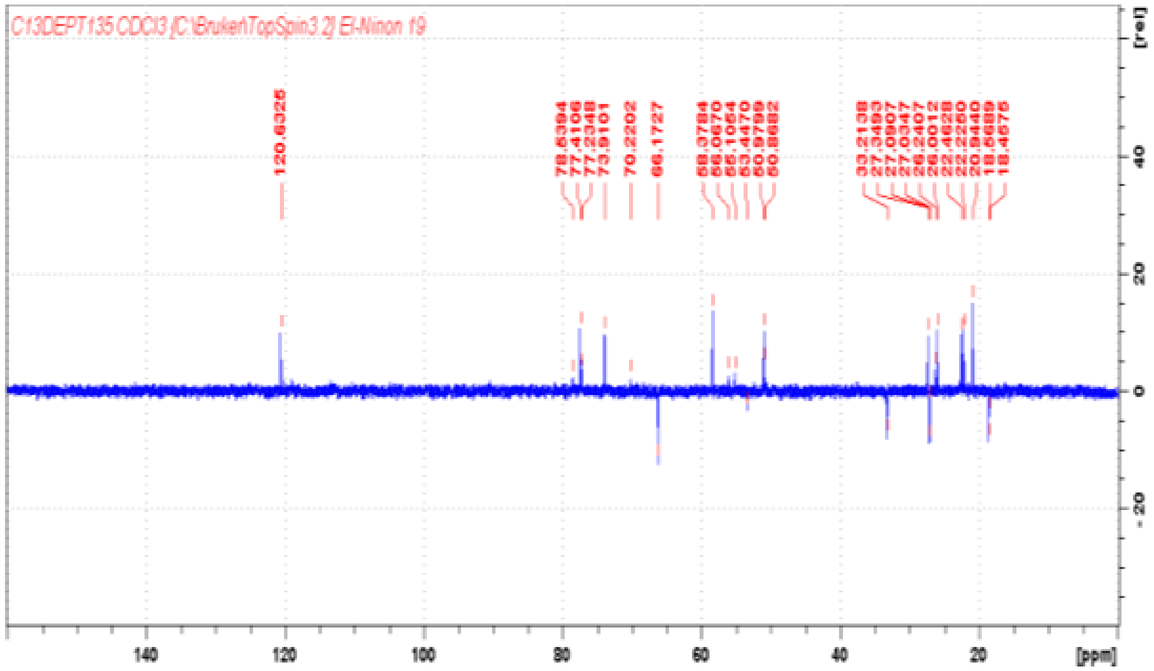

Figure 23: DEPT-NMR (400 MHz, CDCl3) Spectrum of Compound **3**

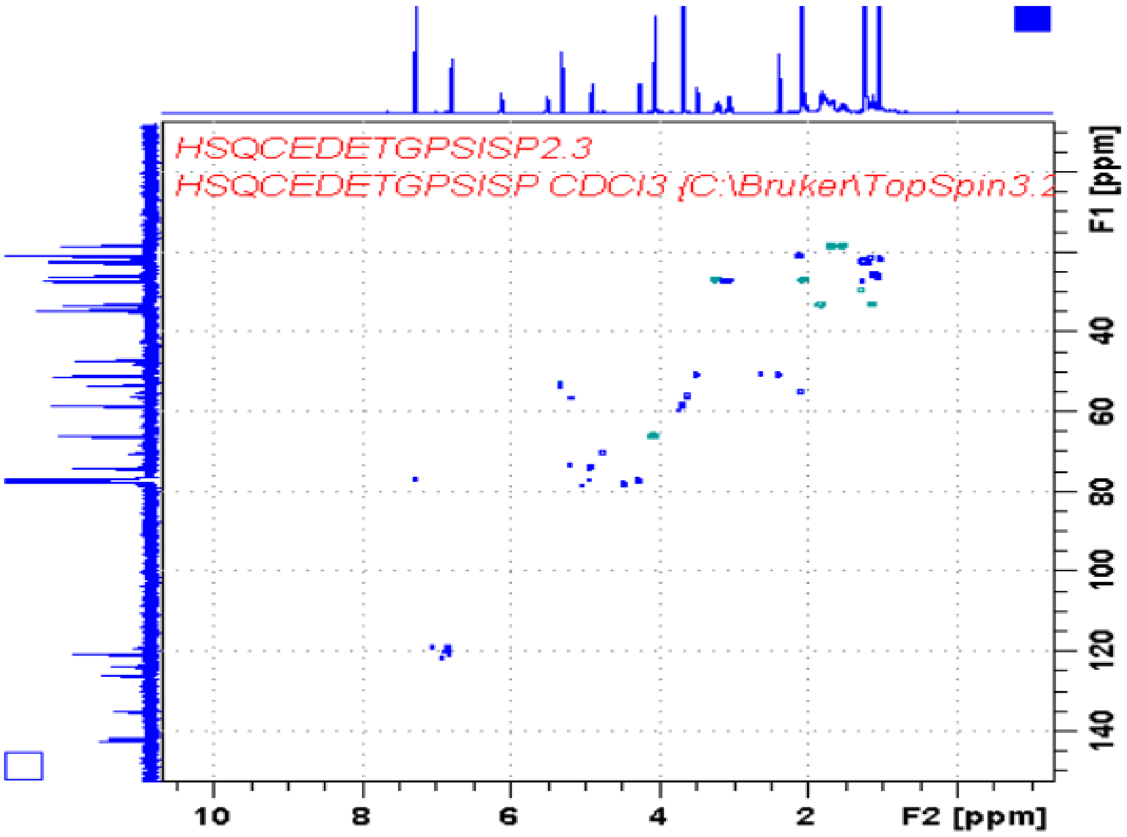

Figure 24: HSQC (400 MHz, CDCl3) Spectrum of Compound **3**

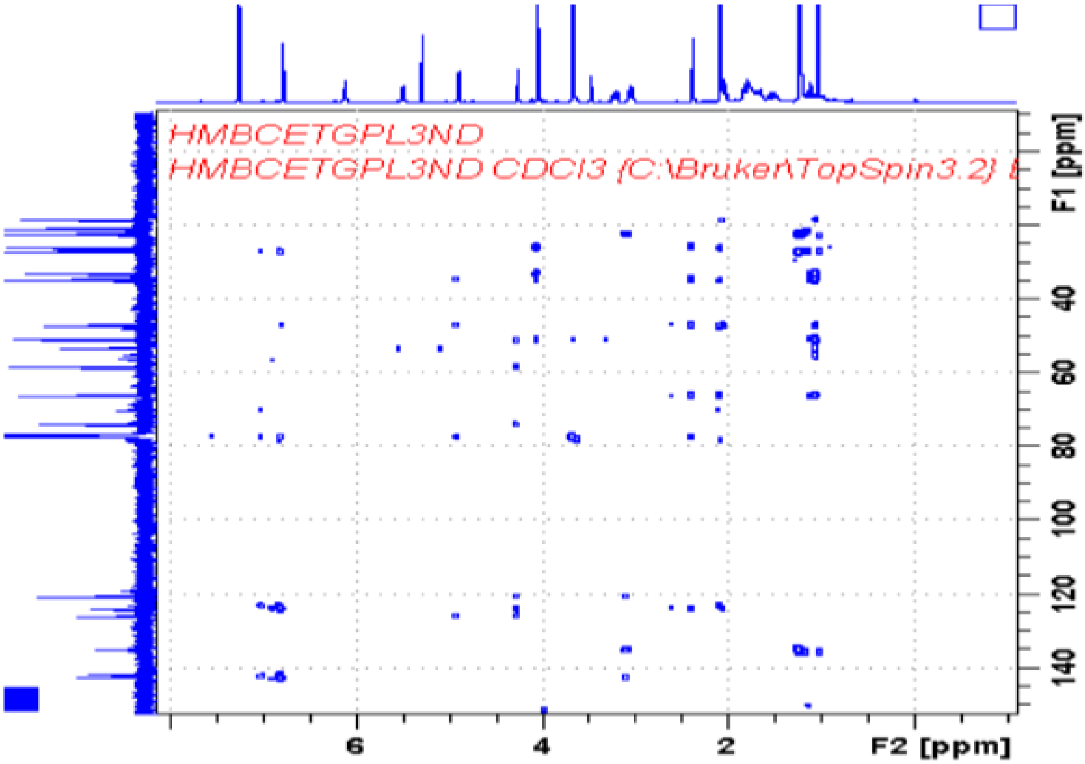

Figure 25: HMBC (400 MHz, CDCl3) Spectrum of Compound **3**

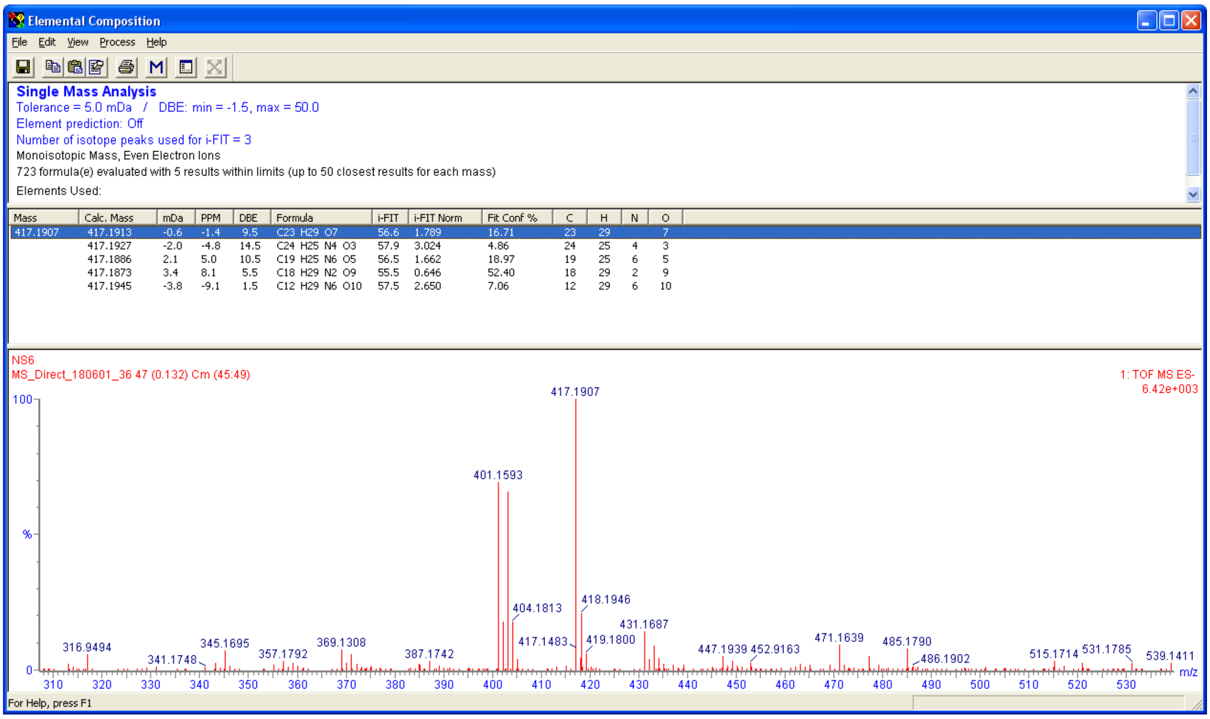

Mass	Calc. Mass	mDa	PPM	DBE	Formula	i-FIT	i-FIT Norm	Fit Conf %	C	H	N	O
417.1907	417.1913	-0.6	-1.4	9.5	C23 H29 O7	56.6	1.789	16.71	23	29		7
	417.1927	-2.0	-4.8	14.5	C24 H25 N4 O3	57.9	3.024	4.86	24	25	4	3
	417.1886	2.1	5.0	10.5	C19 H25 N6 O5	56.5	1.662	18.97	19	25	6	5
	417.1873	3.4	8.1	5.5	C18 H29 N2 O9	55.5	0.646	52.40	18	29	2	9
	417.1945	-3.8	-9.1	1.5	C12 H29 N6 O10	57.5	2.650	7.06	12	29	6	10

Figure 26: HR-MS spectrum of Compound **3**

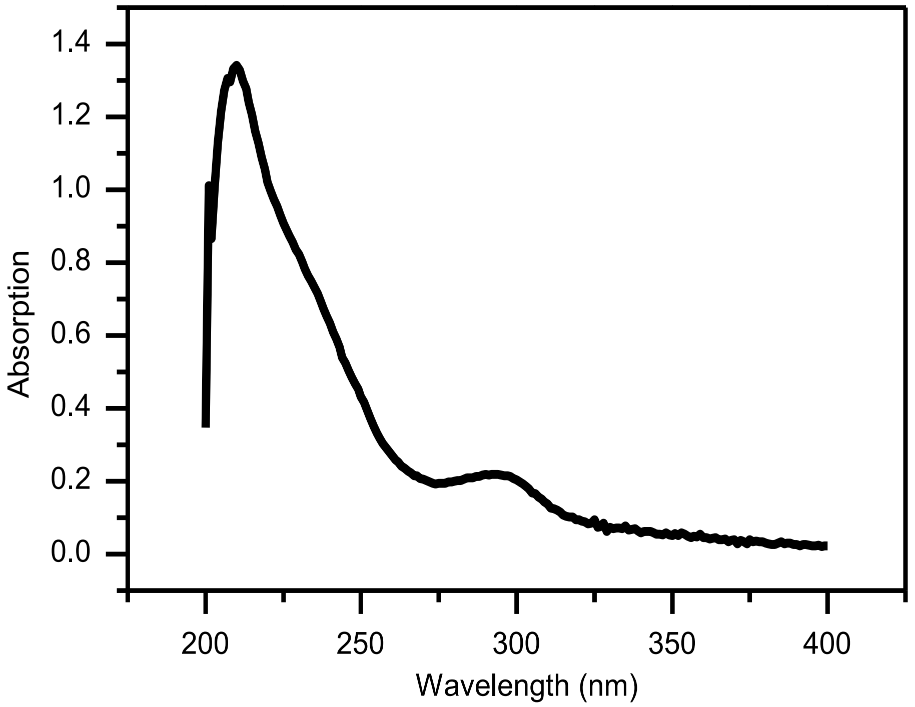

Figure 27: UV (400 MHz, CDCl3) Spectrum of Compound **3**

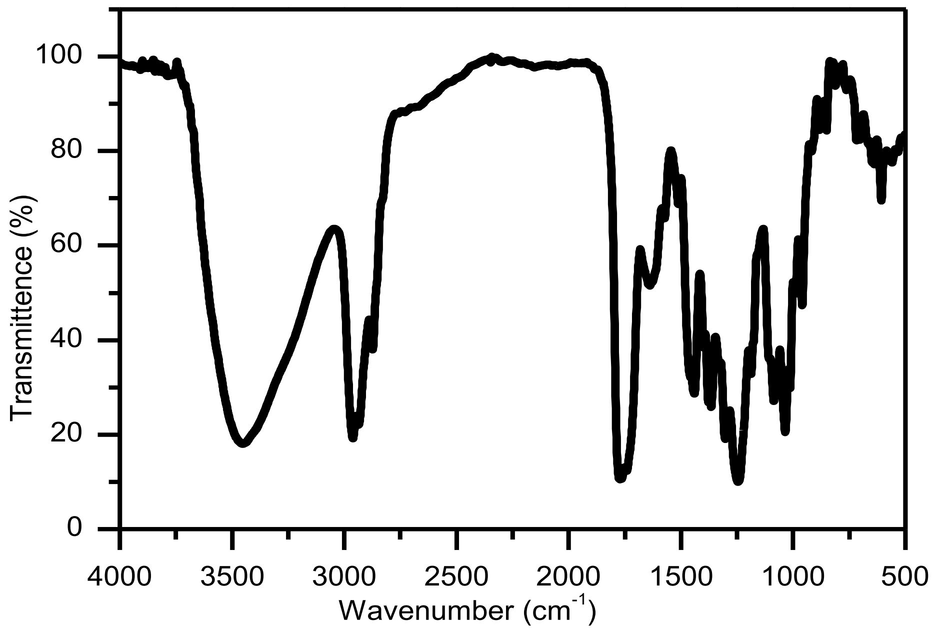

Figure 28: FTIR spectrum of Compound **3**

Compound 4

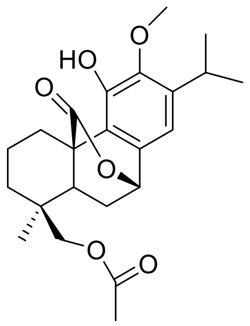

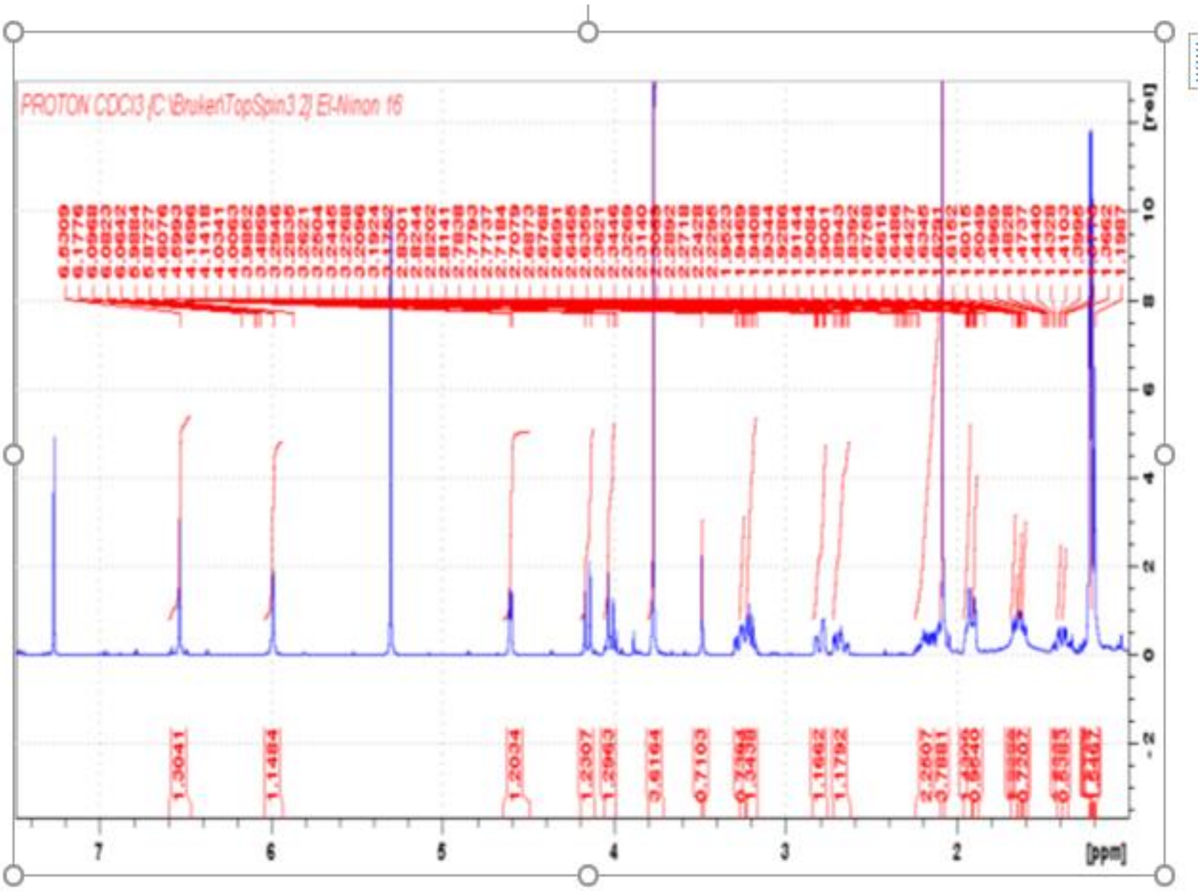

Figure 29: ^{1}H-NMR (400 MHz, CDCl3) Spectrum of Compound **4**

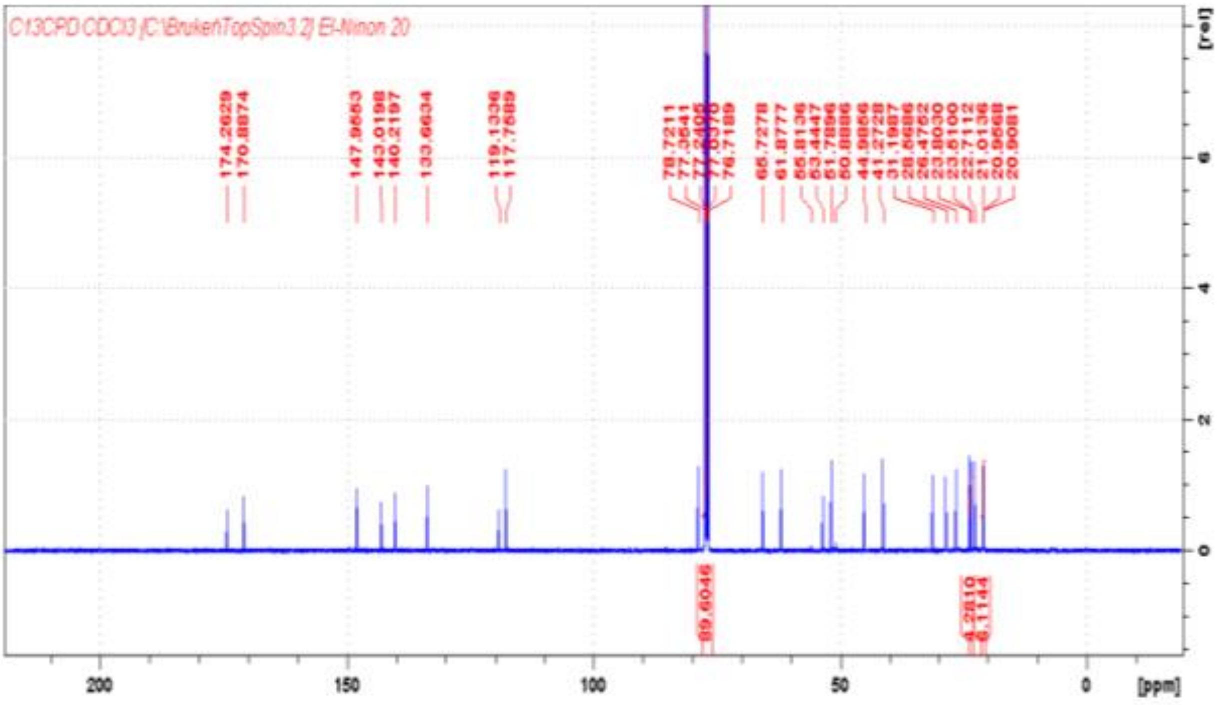

Figure 30: ^{13}C-NMR (400 MHz, CDCl3) Spectrum of Compound **4**

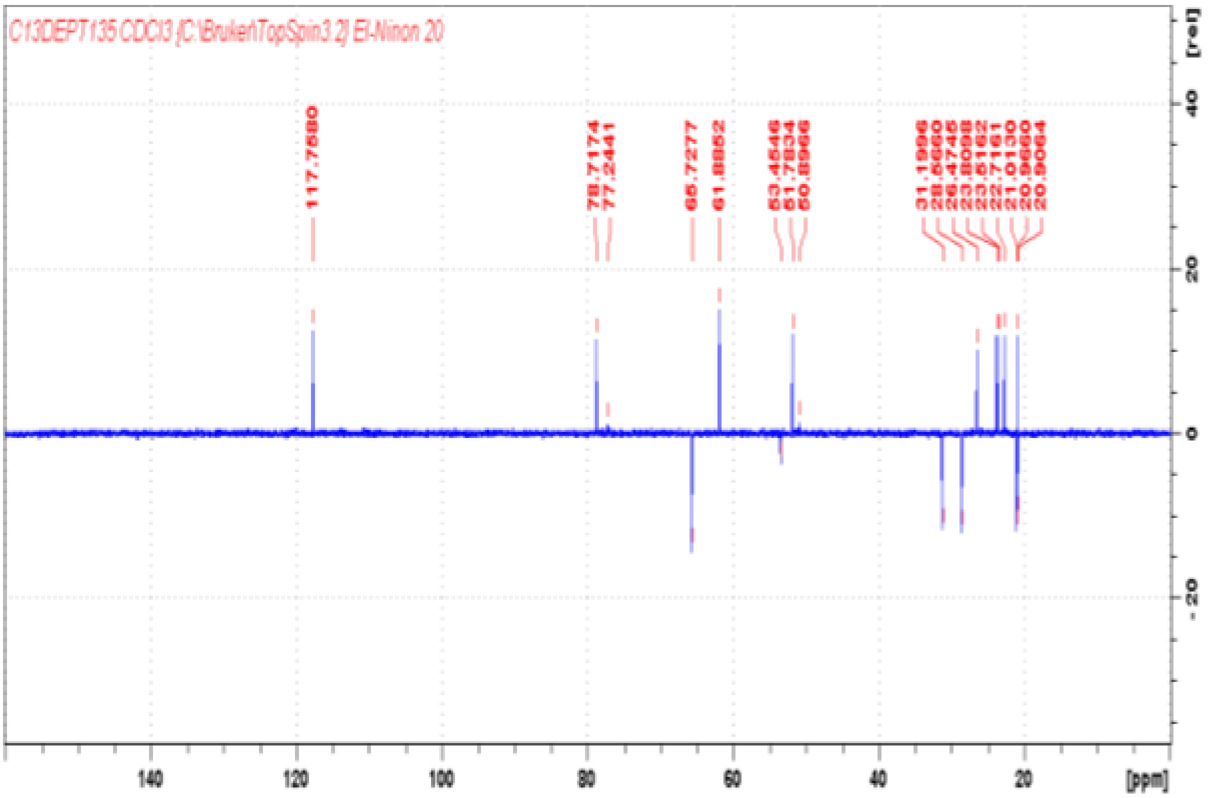

Figure 31: DEPT-NMR (400 MHz, CDCl3) Spectrum of Compound **4**

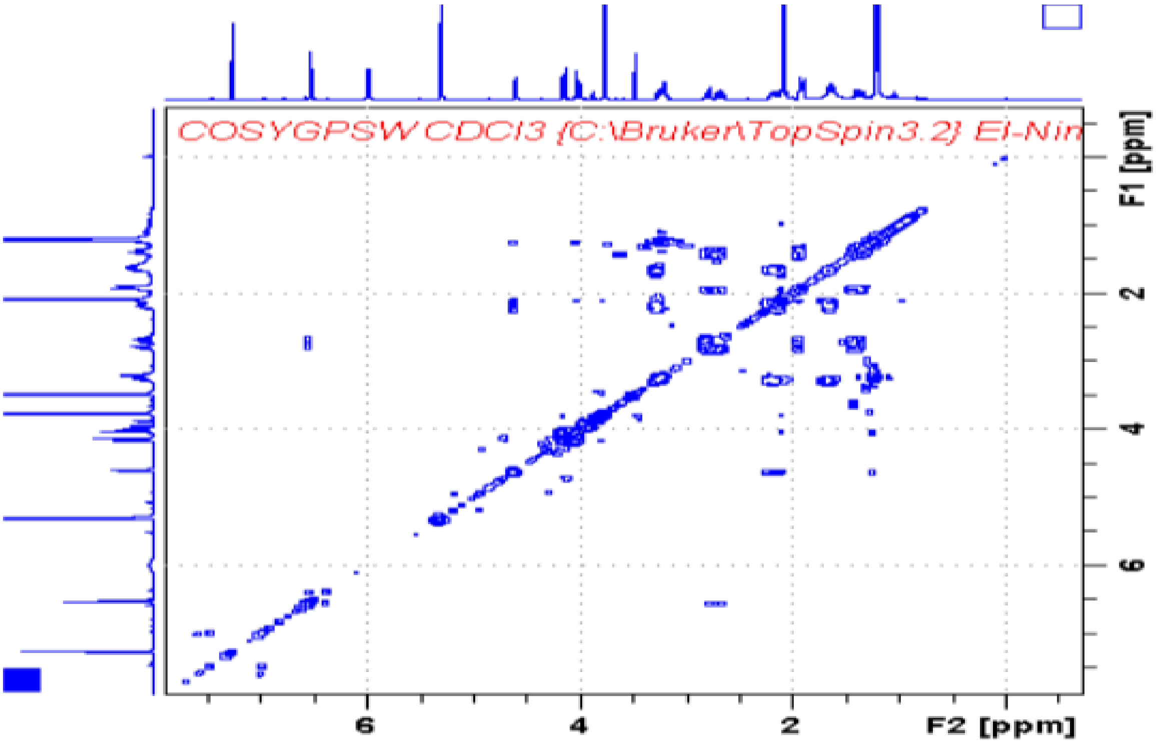

Figure 32: COSY (400 MHz, CDCl3) Spectrum of Compound **4**

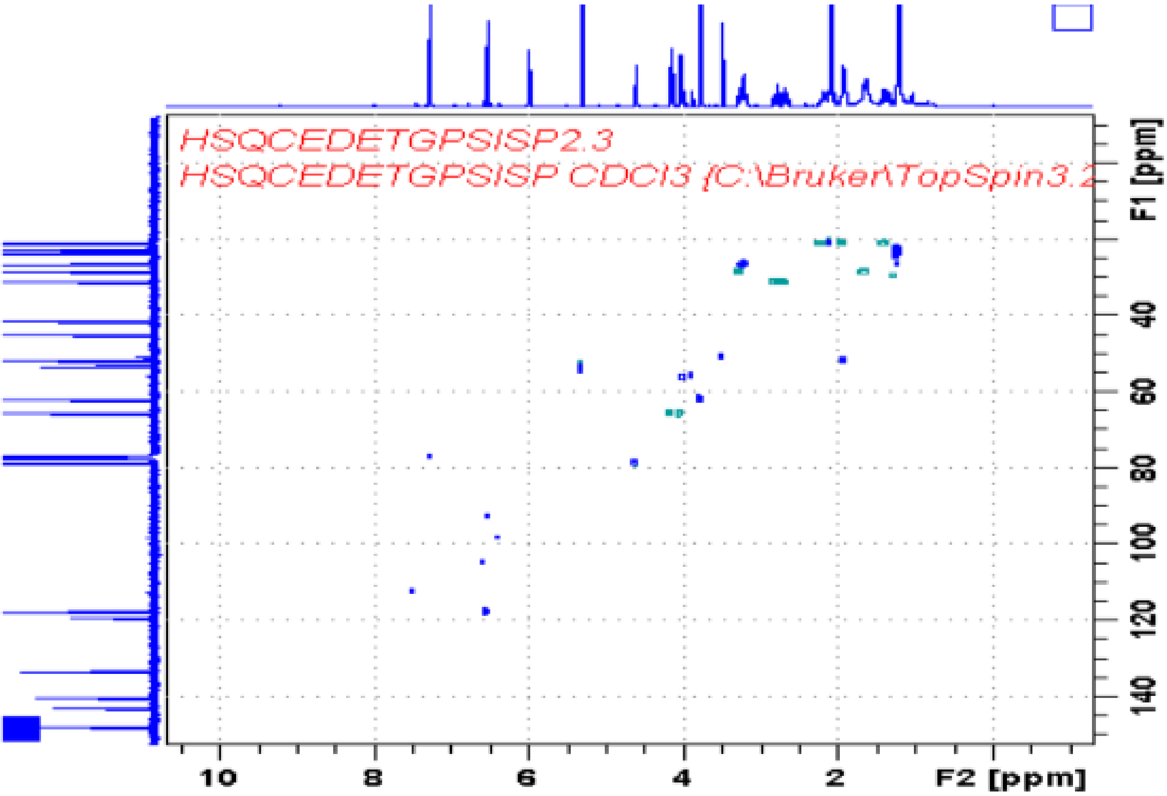

Figure 33: HSQC (400 MHz, CDCl3) Spectrum of Compound **4**

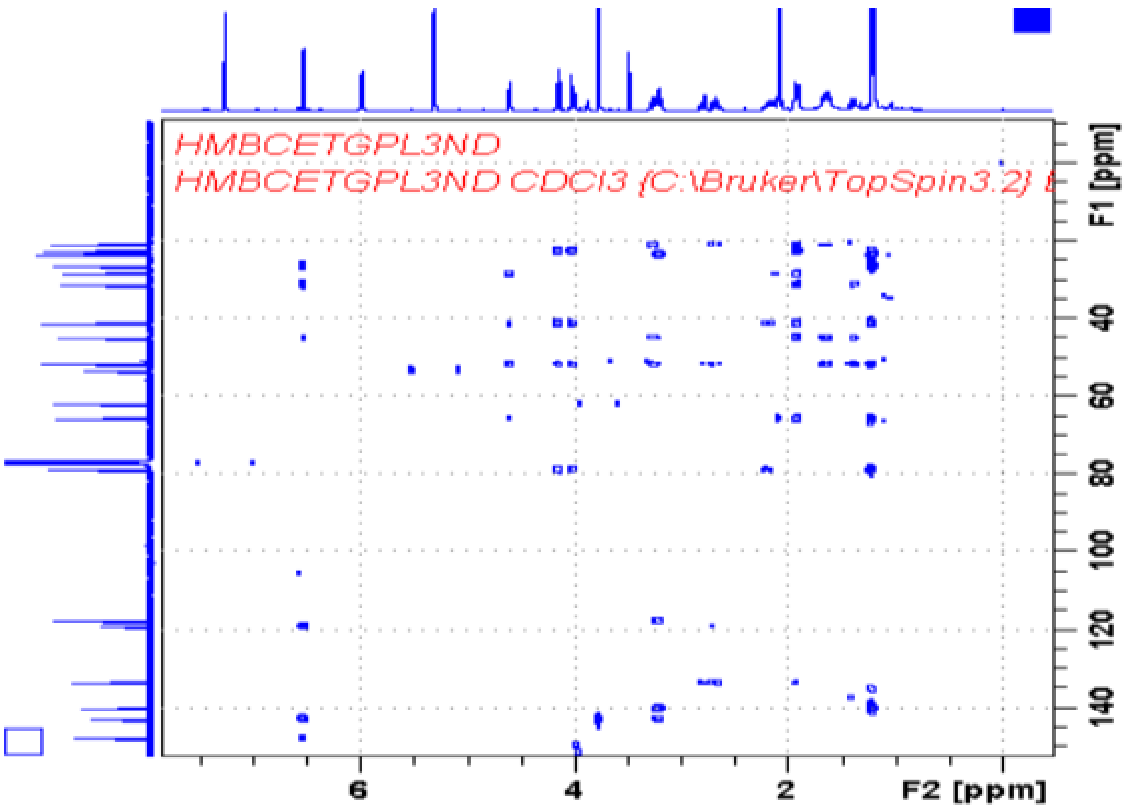

Figure 34: HMBC (400 MHz, CDCl3) Spectrum of Compound **4**

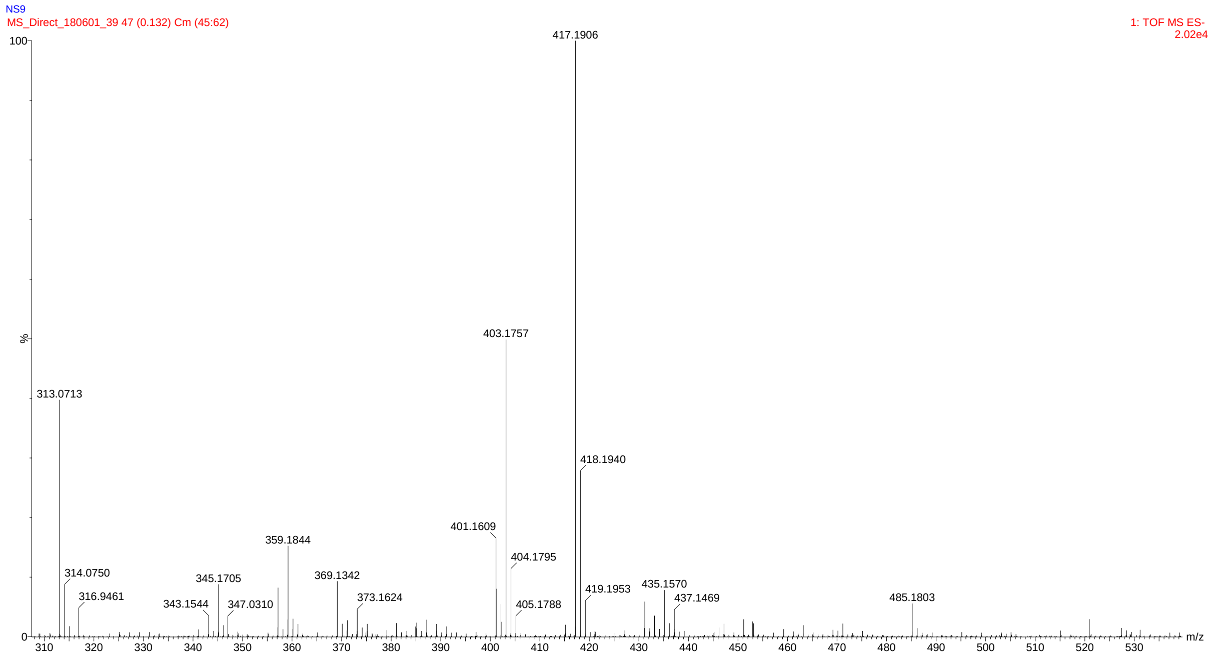

Figure 35: HR-MS spectrum of Compound **4**

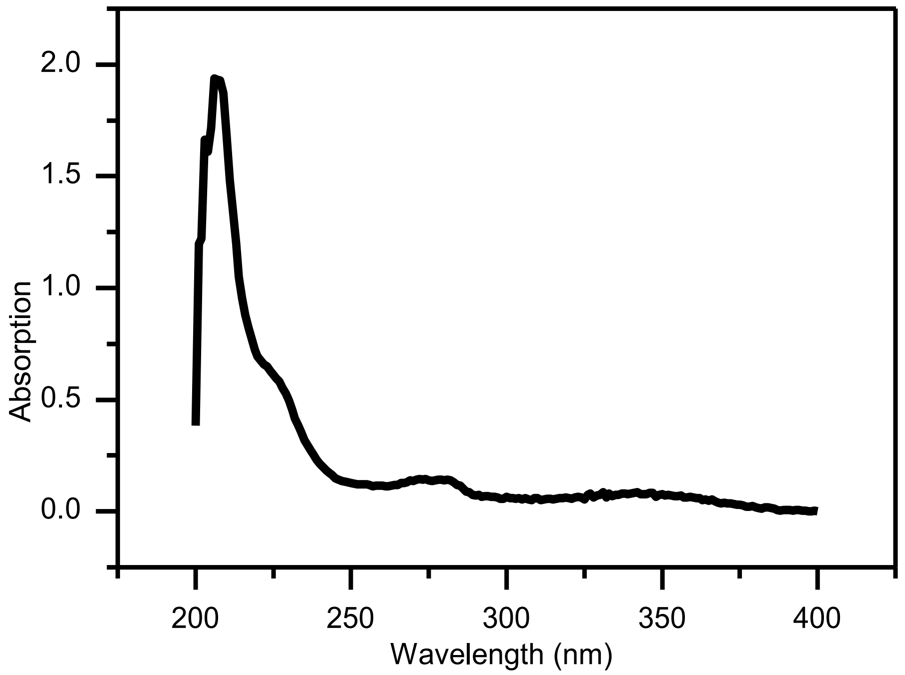

Figure 36: UV spectrum of Compound **4**

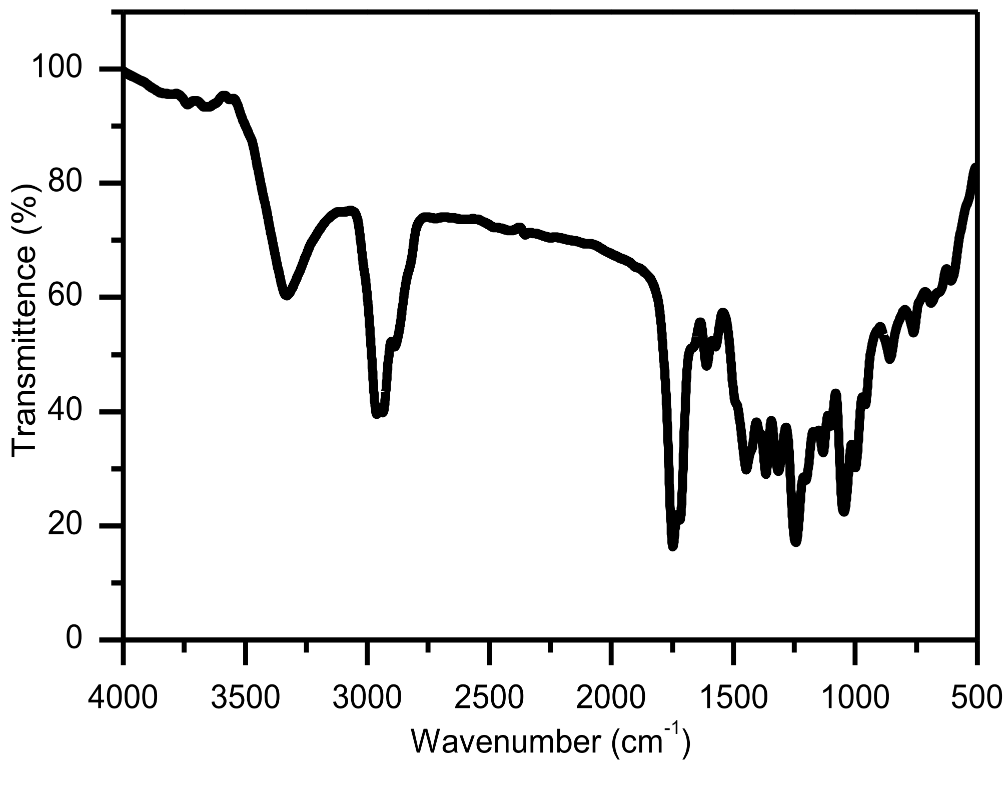

Figure 37: FTIR spectrum of Compound **4**
